# Interactions among ryanodine receptor isotypes contribute to muscle fiber type development and function

**DOI:** 10.1242/dmm.038844

**Published:** 2019-09-18

**Authors:** Alexis A. Chagovetz, Dana Klatt Shaw, Erin Ritchie, Kazuyuki Hoshijima, David J. Grunwald

**Affiliations:** Department of Human Genetics, University of Utah, Salt Lake City, UT 84112, USA

**Keywords:** Ryanodine receptors, Congenital myopathy, Zebrafish disease model, Muscle development, Muscle function

## Abstract

Mutations affecting ryanodine receptor (RyR) calcium release channels commonly underlie congenital myopathies. Although these channels are known principally for their essential roles in muscle contractility, mutations in the human *RYR1* gene result in a broad spectrum of phenotypes, including muscle weakness, altered proportions of fiber types, anomalous muscle fibers with cores or centrally placed nuclei, and dysmorphic craniofacial features. Currently, it is unknown which phenotypes directly reflect requirements for RyRs and which result secondarily to aberrant muscle function. To identify biological processes requiring RyR function, skeletal muscle development was analyzed in zebrafish embryos harboring protein-null mutations. RyR channels contribute to both muscle fiber development and function. Loss of some RyRs had modest effects, altering muscle fiber-type specification in the embryo without compromising viability. In addition, each RyR-encoding gene contributed to normal swimming behavior and muscle function. The RyR channels do not function in a simple additive manner. For example, although isoform RyR1a is sufficient for muscle contraction in the absence of RyR1b, RyR1a normally attenuates the activity of the co-expressed RyR1b channel in slow muscle. RyR3 also acts to modify the functions of other RyR channels. Furthermore, diminished RyR-dependent contractility affects both muscle fiber maturation and craniofacial development. These findings help to explain some of the heterogeneity of phenotypes that accompany RyR1 mutations in humans.

## Abstract (precis)

**Summary:** Skeletal muscle fiber development and function are dependent on additive as well as combinatorial interactions among ryanodine receptor calcium release channels.

## INTRODUCTION

Ryanodine receptor (RyR) intracellular calcium (Ca^2+^) release channels are giant (>2 MDa) homotetrameric ion channels that mediate release of Ca^2+^ from intracellular stores (Fill and Copello, 2002; Lanner et al., 2010). In humans, RYR1 channels are best known for their role in excitation–contraction coupling (ECC), which links motor neuron signaling to skeletal muscle contractions (Fill and Copello, 2002; Treves et al., 2017) and mutations in the *RYR1* gene are a major causative factor of many congenital myopathies (Jungbluth et al., 2018; Klein et al., 2012; Sewry et al., 2008). During ECC, activation of acetylcholine receptors leads to depolarization of dihydropyridine receptors at the surfaces of muscle cells, producing conformational changes in the tightly associated RyR1 channels, thus triggering release of Ca^2+^ from the sarcoplasmic reticulum and contraction of skeletal muscle fibers (Fambrough, 1979; Protasi, 2002; Tanabe et al., 1988). Nevertheless, several findings indicate that RyR channel activity is not limited to its role in ECC. First, intracellular Ca^2+^ mobilization is a required intermediary in many kinds of intracellular signaling cascades, including those involved in cell motility (Matyash et al., 2002), transcription regulation (Buonanno and Fields, 1999), apoptosis (Hajnóczky et al., 2000) and other essential cellular processes (Berridge et al., 2003). Second, RyR channels are expressed in noncontractile tissues including the kidney, lymphocytes, cerebellum, ovary and testis (Marks et al., 1989; Nakai et al., 1990; Takeshima et al., 1989). Third, RyR function has been tied to organelle mobilization, sensitivity to intercellular signaling and memory formation (Baker et al., 2013; Collin et al., 2005; Klatt Shaw et al., 2018; Liu et al., 2005). Furthermore, mutations in the *RYR1* gene affect muscle fiber size and structure as well as fiber type predominance (Clarke et al., 2010; Treves et al., 2008), raising the possibility that RyR channels have roles in processes contributing to skeletal muscle development.

Indeed, a wide range of clinical phenotypes have been associated with the congenital myopathies resulting from mutations in *RYR1*, the RyR-encoding gene predominantly expressed in human skeletal muscle (Abath Neto et al., 2017; Bharucha-Goebel et al., 2013; Clarke et al., 2010; D'Arcy et al., 2008; Dowling et al., 2011; Jungbluth et al., 2012; Klein et al., 2012; Snoeck et al., 2015; Treves et al., 2008; Wei and Dirksen, 2010; Wilmshurst et al., 2010). Mutations have been associated with a heterogeneous assortment of contractile, physiological and developmental defects. Although most individuals with *RYR1* mutations suffer proximal muscle weakness, many also exhibit weakness of respiratory and/or extraocular muscles, and some individuals are primarily affected in distal muscles (Klein et al., 2012; Laughlin et al., 2017). A particular set of *RYR1* alleles renders the channels leaky upon administration of anesthetic agents, leading to a life-threatening condition called malignant hyperthermia (Maclennan and Zvaritch, 2011; Rosenberg et al., 2015). A potentially related set of alleles causes exercise-triggered rhabdomyolysis (Dlamini et al., 2013). A separate set of recessive loss-of-function and dominant alleles result in congenital myopathies characterized by formation of histologically recognized cores that often lack mitochondria (Amburgey et al., 2013; Jungbluth et al., 2011; Treves et al., 2008). Individuals with *RYR1* mutations often display additional structural anomalies in their fibers, including fiber size irregularity, a condition known as congenital fiber type disproportion (CFTD), and a predominance of type I (slow) fibers (Clarke et al., 2010; Sato et al., 2008). Other mutations are noted for resulting in abnormal centrally located nuclei in skeletal muscle fibers (Abath Neto et al., 2017; Jungbluth et al., 2007; Wilmshurst et al., 2010). Genotypes that clearly bring about loss of function are associated with severe disease presentation and are enriched for centronuclear myopathy and CFTD phenotypes as well as ophthalmoparesis (Amburgey et al., 2013). Severely affected individuals exhibit skeletal abnormalities, frequently including an arched palate and additional distinctive craniofacial features (Bharucha-Goebel et al., 2013; D'Arcy et al., 2008; Dowling et al., 2011; Snoeck et al., 2015). The phenotypic heterogeneity associated with skeletal muscle *RYR1* mutations in humans makes it difficult to deduce the immediate defects that result from altered RyR channel function.

Several factors limit the insights that clinical characterizations can provide into the functions of RyR channels in developing muscle. First, human phenotypes are generally recognized postnatally and thus the observed muscle anomalies probably result from a combination of effects on development, function and regeneration. Second, human studies are confounded by both extensive genetic heterogeneity and characterizations that could be limited by biopsied tissue or that focus on subsets of a phenotype (Kushnir et al., 2018; Maclennan and Zvaritch, 2011; Treves et al., 2008). Furthermore, as multiple RyR channels can be expressed in vertebrate skeletal muscle, it is possible that some mutant alleles affect interactions among the channels that could complicate interpretation of mutant phenotypes (Yang et al., 2001).

To help identify the essential roles of RyR channels in the initial establishment of muscle, we studied the phenotypic consequences of RyR loss of function in the zebrafish myotome. Development of somitic muscle has been especially well characterized in the zebrafish embryo (Jackson and Ingham, 2013; Stickney et al., 2000). There are two general muscle fiber types in vertebrates: slow-twitch (slow) and fast-twitch (fast) (Schiaffino and Reggiani, 2011). Slow fibers exhibit slow contraction velocities and have abundant mitochondria, which result in fibers that are resistant to fatigue. Fast fibers are utilized to generate short high-speed bursts, but fatigue rapidly. Unlike mammalian muscle, slow and fast muscle fibers are physically segregated in fish (Stickney et al., 2000), which allowed us to address how individual RyRs contribute to the development and function of skeletal muscle fiber types.

Zebrafish have five genes encoding RyR isoforms (this family of genes is referred to hereafter as ryr genes), three of which are expressed in skeletal muscle: *ryr1a* is expressed only in slow fibers, *ryr3* expression appears limited to fast muscle and *ryr1b* is expressed in both slow and fast fibers [(Wu et al., 2011) and this study]. The *ryr1a* and *ryr1b* genes are closely related paralogs of the ancestral *ryr1* gene, which experienced a copy number expansion in zebrafish as a consequence of the teleost-specific whole genome duplication (Howe et al., 2013; Postlethwait et al., 1998). The distinct expression patterns of each gene within the myotome enabled us to ask whether RyR channels are differentially required for slow and fast fiber development and/or function.

Here, we present the phenotypic consequences of eliminating each of the RyR channels expressed in the developing skeletal muscle of zebrafish. Only one mutant affecting any of the zebrafish ryr genes has been described previously. The *relatively relaxed* mutation is probably a hypomorphic allele that arose spontaneously in the *ryr1b* gene (Hirata et al., 2007). Study of *relatively relaxed* indicated that the RyR1b channel was required for Ca^2+^ release in fast but not slow fibers during contraction, indicating that RyR channels have fiber type-specific roles in zebrafish skeletal muscle. However, given the nature of the allele used for the study and the focus on only one RyR channel, it remains unclear whether the RyR channels present in the zebrafish myotome have distinct or overlapping roles.

We generated complete loss-of-function alleles for each zebrafish ryr gene expressed in the myotome and determined the tissue-specific roles of individual RyR channels using a suite of genetic, immunohistochemical and behavioral assays. First, each RyR channel contributes to muscle activity and swimming behavior. Second, loss of RyR activity affects the embryonic process of muscle fiber-type specification, producing mild defects that alter fiber type predominance without necessarily compromising viability. In the zebrafish, RyR1a and RyR1b have fiber-specific contributions: both are required for slow fiber activity, whereas only RyR1b is necessary for fast fiber activity. Our findings reveal that co-expressed RyR channels do not function independently of one another. Rather, RyR1a and RyR1b channels contribute in a nonadditive manner to Ca^2+^ mobilization and muscle contraction. Similarly, although RyR3 is not required for muscle contraction, it modifies contractile behavior. Our results indicate that RyR-dependent muscle contraction contributes to proper maturation of muscle fibers and formation of jaw morphology during early development, phenotypes observed in subsets of individuals with *RYR1* mutations (Bharucha-Goebel et al., 2013; Jungbluth et al., 2012; Sato et al., 2008).

## RESULTS

### Mutant ryr alleles do not produce protein products

Three ryr genes, *ryr1a*,* ryr1b* and *ryr3*, are expressed in the myotome of the zebrafish embryo (Wu et al., 2011). We generated loss-of-function mutations in *ryr1a*,* ryr1b* and *ryr3* to uncover their functions in embryogenesis. Individual genes were targeted with TALENs to induce indel mutations in coding sequences (Dahlem et al., 2012). Because each ryr gene has over 100 exons and is subject to extensive alternate splicing, we chose to target exon 6 for mutagenesis, because it is an early exon that is included in all known ryr splice variants (Flicek et al., 2011). For each ryr gene expressed in zebrafish muscle, alleles harboring premature stop codons that were predicted to truncate the protein products severely were recovered (Fig. S1). As presumed null alleles can be expressed due to exon skipping (Anderson et al., 2017; Lalonde et al., 2017; Mou et al., 2017), we tested whether the mutant ryr alleles were truly protein null.

**Fig. 1.**
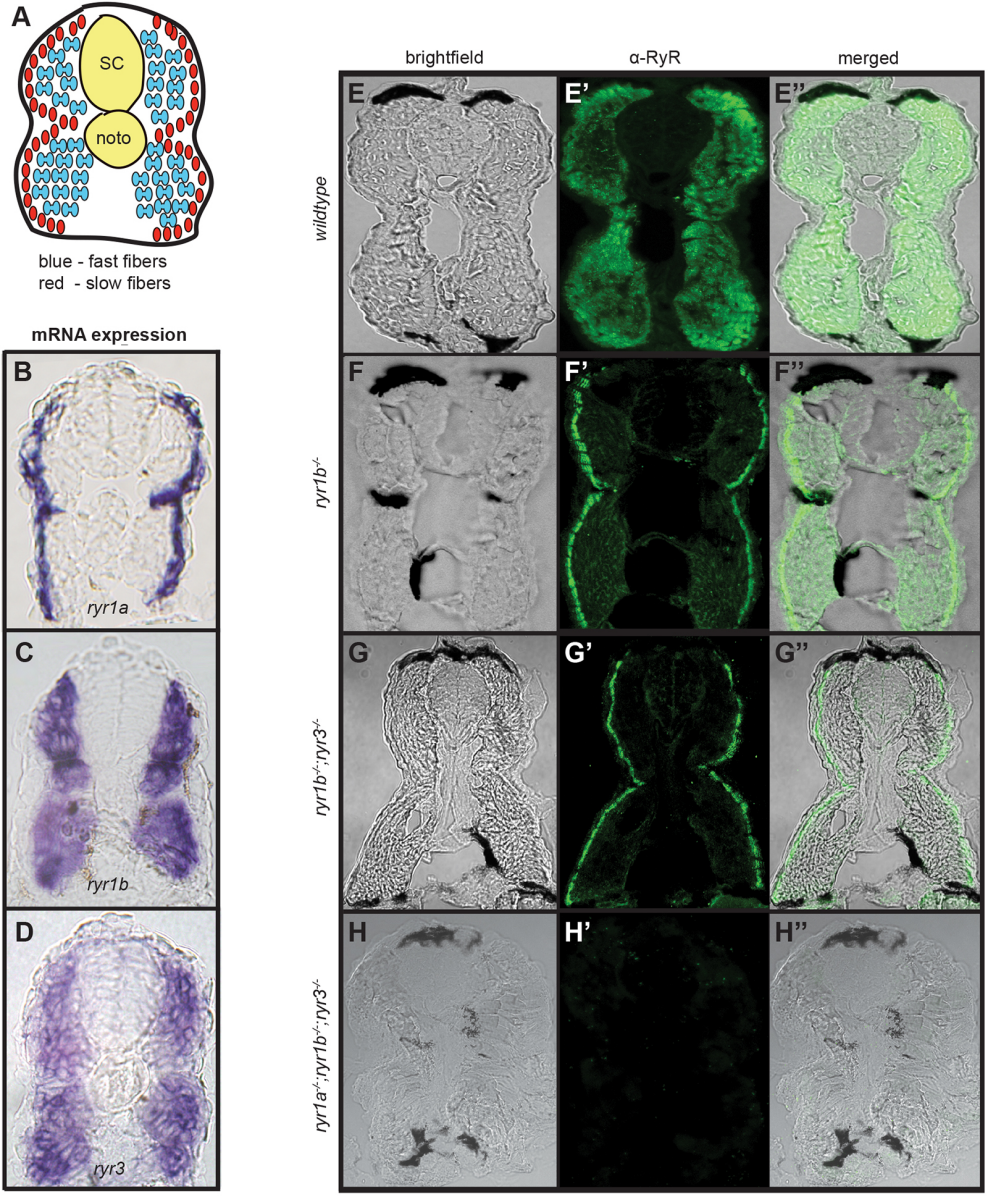
**Mutant ryr alleles do not produce protein products.** (A) Schematic of a cross-section of the trunk of a 1-2 dpf zebrafish embryo highlighting muscle organization. SC spinal cord, noto notochord. (B-D) Transverse cryosections of 24 hpf embryo trunks illustrating the RNA expression patterns of (B) *ryr1a*, (C) *ryr1b*, and (D) *ryr3* detected by WISH. (E-H″) Transverse sections through the trunks of 48 hpf wild-type and mutant embryos immunostained with the 34C (anti-RyR) antibody. Brightfield images (E-H), fluorescent images showing 34C staining (green) (E′-H′), and merged brightfield/fluorescent images (E″-H″) of embryos of indicated genotypes. Each ryr mutation is associated with loss of a distinct component of the normal expression pattern of RyR channels in embryonic muscle. Somitic muscle of triple *ryr1a;ryr1b;ryr3* mutants lack all RyR protein detected by the 34C antibody.

Within the zebrafish trunk, slow muscle fibers occupy a superficial single-cell layer near the dermis, whereas fast muscle fibers make up most of the deeper layers (Fig. 1A). Collectively, transcripts of the three genes (*ryr1a*,* ryr1b* and *ryr3*) are present in both superficial and deep muscle in 1 day post fertilization (dpf) wild-type embryos (Fig. 1B-D). RyR protein products were visualized in transverse cross-sections of trunks of 2 dpf zebrafish embryos using the pan-RyR 34C monoclonal antibody (Airey et al., 1990). RyR channels were present in both superficial slow and deep fast fibers of each somite (Fig. 1E-E″). In *ryr1b^z43^* mutant embryos, staining in fast muscle was decreased, but not absent (Fig. 1F-F″). Because *ryr3* transcripts are also expressed in fast muscle (Fig. 1D), we hypothesized that the RyR3 channels were responsible for the remaining signal in the deep myotome. Indeed, expression of RyR proteins in fast fibers was totally lost in *ryr1b^z43^;ryr3^z45^* embryos (Fig. 1G-G″), indicating that the two isotypes are the only RyR channels present in fast fibers. The remaining RyR signal in the slow fibers of *ryr1b^z43^;ryr3^z45^* embryos was due to expression of *ryr1a* in these cells (Fig. 1B), as all RyR signal was absent from the myotomes of *ryr1a^z42^;ryr1b^z43^;ryr3^z45^* triple mutants (Fig. 1H-H″). Consistent with the findings that each mutant allele failed to produce protein product, whole-mount *in situ* hybridization (WISH) revealed that *ryr1a* and *ryr1b* mutants had reduced steady-state amounts of their respective mRNAs (Fig. S2), as might occur as a result of nonsense-mediated decay (Conti and Izaurralde, 2005). Based on these data, we concluded that the mutations were protein-null alleles.

The null mutants have helped clarify the expression patterns of the three genes. Expression of *ryr1a* was restricted to superficial slow muscle fibers (Fig. 1B). Although *ryr3* is maternally supplied (Klatt Shaw et al., 2018) and transcripts can be detected diffusely at somitogenesis stages (Jurynec et al., 2008; Wu et al., 2011), *ryr3* transcripts appear predominantly in fast fibers at 1 dpf (Fig. 1D). In contrast, *ryr1b* is expressed in both fast and slow fibers of embryos. The *ryr1b* transcripts are evident in the deep muscle of 1 dpf embryo somites (Fig. 1C). At 1 and 2 dpf, RyR1b protein appears to be the predominant RyR isotype in fast muscle, as revealed by immunohistochemistry staining of mutants lacking RyR3 channels (Fig. 1E–G, Fig. S3A,C). In addition, *ryr1b* transcripts were conspicuous at early somitogenesis stages in the adaxial cells (Fig. S3E), the immediate progenitors of slow muscle fibers (Devoto et al., 1996). Furthermore, RyR1b protein was present in the superficial slow muscle fibers of 1 dpf embryos devoid of RyR1a and RyR3 channels (Fig. S3B,D). These results are consistent with previous characterizations of ryr transcript expression in zebrafish (Wu et al., 2011).

### Muscle patterning is perturbed in ryr mutant embryos

Having verified each mutant allele as being protein null, we determined whether ryr genes were essential for normal zebrafish development. Homozygous *ryr1a*, *ryr3* and double *ryr1a;ryr3* mutants survive to adulthood and produce viable fertile offspring, indicating that muscle development and function is not severely compromised despite loss of the RyR1a and RyR3 channels. In contrast, *ryr1b* animals are poor swimmers, fail to inflate swim bladders and do not reach adulthood, in agreement with a study of the *relatively relaxed* mutant (Hirata et al., 2007). Additionally, consistent with mouse studies, complete loss of *ryr1* function results in loss of skeletal muscle contractility. Zebrafish *ryr1a;ryr1b* double-mutant embryos are completely paralyzed, fail to inflate swim bladders and die at ∼7 dpf (see below). The *ryr1a;ryr1b;ryr3* triple mutants exhibit the same lethal locomotor phenotype as the *ryr1a;ryr1b* mutants (data not shown).

**Fig. 2.**
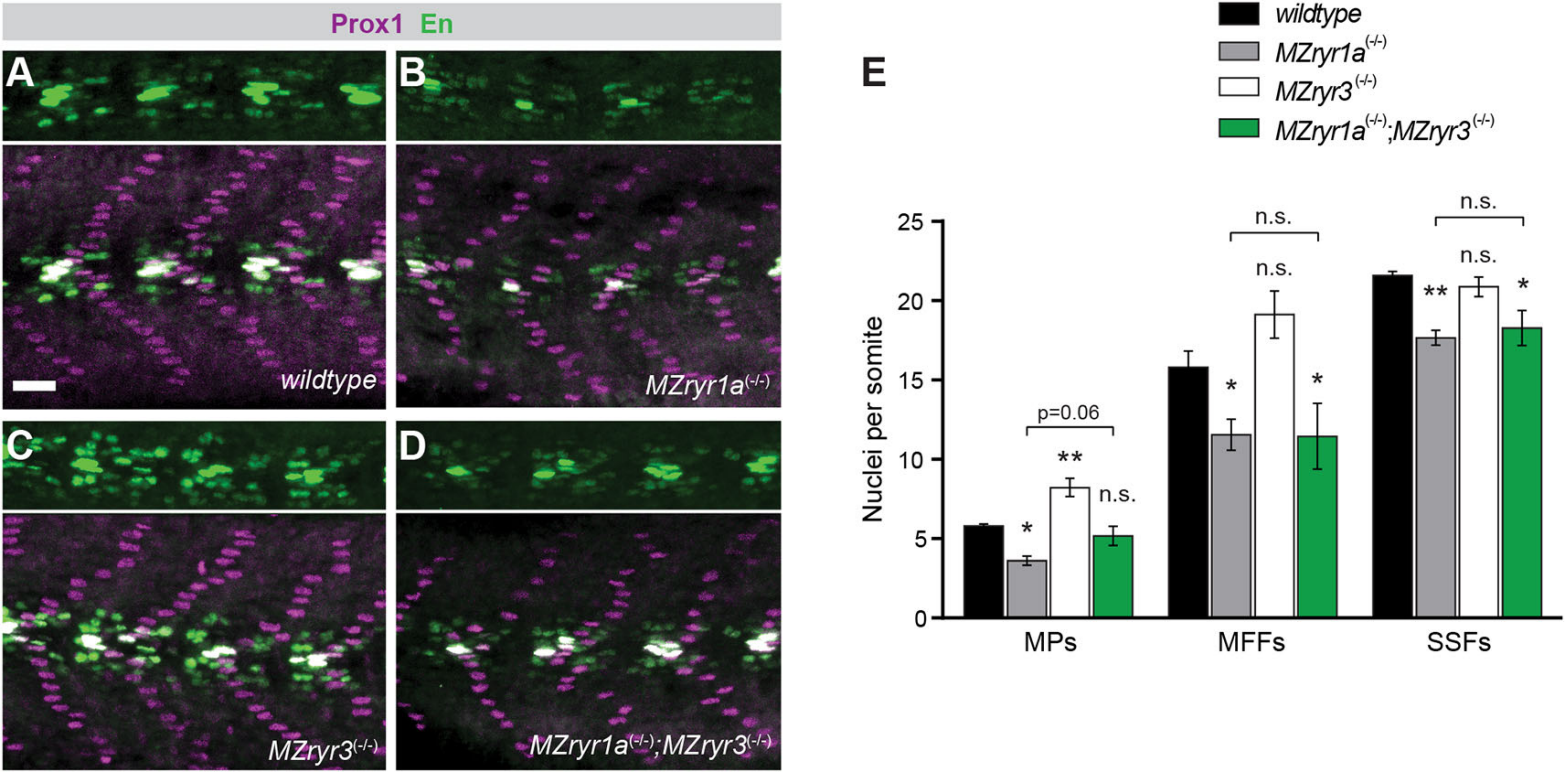
**Formation of Shh-dependent muscle in ryr mutants.** Muscle cell type patterning in wild-type and mutant zebrafish 24 hpf embryos was assessed by immunohistochemical staining for expression of the Prox1 and Engrailed nuclear proteins. (A-D) Representative images of Prox1 (magenta) and Engrailed (green) staining of somitic muscle in wild-type (A), *MZryr1a* (B), *MZryr3* (C) and *MZryr1a;MZryr3* (D) embryos. Slow muscle pioneer cells (MPs) were identified as cells that expressed both Prox1 and Engrailed antigens; medial fast fibers (MFFs) were identified as cells that expressed only the Engrailed antigen; and superficial slow fibers (SSFs) were identified as cells that expressed only the Prox1 antigen. (E) Quantification of Shh-dependent muscle cell types. One-way ANOVA was used to determine statistical relationships with Sidak's multiple comparisons test used to adjust *P*-values. n.s., not significant; *P<0.01, **P<0.001.

*RYR1* mutations in humans are often associated with congenital fiber type disproportion and occasionally with type 1 fiber predominance (Bharucha-Goebel et al., 2013; Clarke et al., 2010; Jungbluth et al., 2012; Morrison, 2008; Sato et al., 2008). We investigated whether ryr mutations might affect fiber-type specification in zebrafish without severely diminishing viability. Previously, we showed that near-complete loss of zebrafish RyR activity, resulting from either pharmacologic inhibition or combinations of ryr mutations, dramatically disrupted global Sonic hedgehog (Shh) growth factor signaling, with pleiotropic effects on tissue specification and patterning (Klatt Shaw et al., 2018). As noted above, zebrafish lacking both maternal and zygotic supply of *ryr1a* or *ryr3* are fully viable and display no overt signs of altered Shh patterning. To detect subtle effects on muscle specification in the mutants, we examined the formation of Shh-dependent muscle cell types in mutant embryos (Fig. 2).

**Fig. 3.**
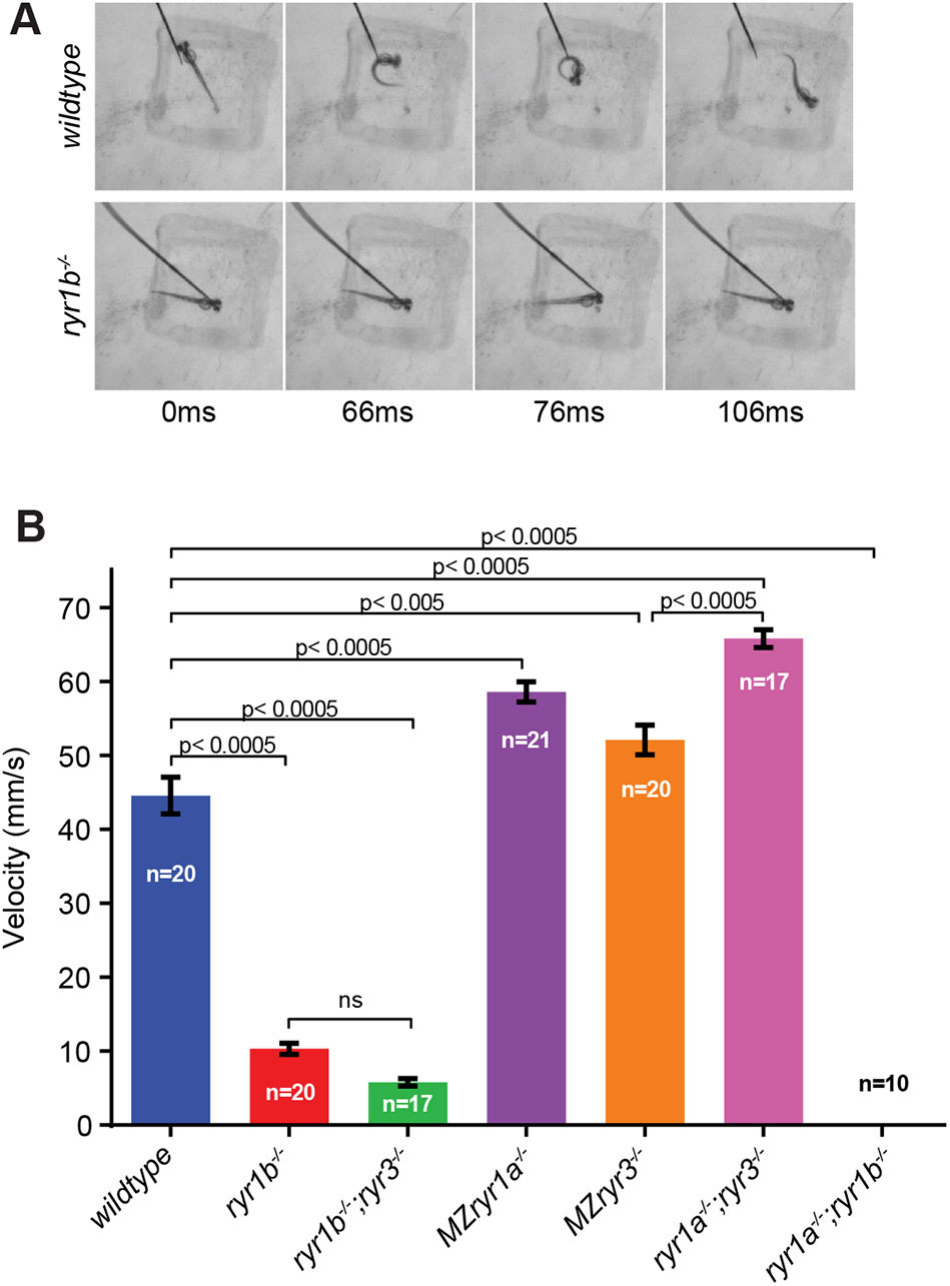
**Single and compound ryr mutants have altered escape velocities.** (A) Still images from high-speed recordings of startle response in 48 hpf larvae taken from Movie 1 (wild-type) and Movie 2 (*ryr1b*). The genotypes of animals are displayed on the left and times after stimulation are displayed along the bottom. The wild-type animal displays characteristic C-bend behavior, whereas the *ryr1b* mutant never displays a contraction of the entire trunk. (B) Escape velocities of 3 dpf wild-type and mutant larvae. The *ryr1b* mutants displayed reduced escape velocity, whereas *ryr1a;ryr1b* double mutants were paralyzed. Loss of RyR1a, RyR3 or both RyR1a and RyR3 channels resulted in larvae that swam faster than wild-type controls. Statistical significance was determined using one-way ANOVA test with Tukey's multiple comparisons test used to adjust the *P*-values. ns, not significant.

Zebrafish mutant *MZryr1a* embryos lacking both maternal and zygotic *ryr1a* function exhibited a significant decrease in the number of Shh-dependent muscle cells (Wolff et al., 2003), including slow muscle pioneer (MP) cells, medial fast fibers (MFF) and superficial slow fibers (SSF) (Fig. 2A,B,E). Loss of only zygotic *ryr1a* function was also sufficient to reduce Shh-dependent muscle development (Fig. S4A,B,D). Remarkably, *MZryr3* mutant embryos displayed a significant increase in the number of MP cells (Fig. 2C,E). However, *MZryr1a;MZryr3* mutant embryos had Shh-dependent muscle specification similar to that of *MZryr1a* mutant embryos (Fig. 2B,D,E). In contrast, despite the severe effect on swimming and contractility (Fig. 3), loss of zygotic *ryr1b* had no measurable effect on Shh-dependent muscle patterning in either wild-type embryos or embryos carrying additional ryr mutations (Fig. S4). To summarize, altered RyR channel activity during skeletal muscle development can be expected to affect fiber-type specification and the effects on specification and contractility or viability can be uncoupled.

### RyR channel function is needed for muscle contraction contributing to both startle response and swimming behaviors

Behavioral analysis was used to study the effects of RyR loss of function on locomotion. Two well-characterized behavioral responses of zebrafish embryos to stimuli were assayed: (1) C-start behavior at 2 dpf and (2) burst swimming velocity at 3 dpf. The C-start is an early component of startle response behavior. In reaction to stimuli, unilateral muscle contractions lead the head and tail to bend toward each other, forming a ‘C-shape,’ after which the fish swims away from the source of the stimulation (Budick and O'Malley, 2000; Kimmel et al., 1974). C-start behavior was initiated via tactile stimulation of the head and recorded using a high-speed camera (500 frames/s). Wild-type animals always displayed C-bends upon stimulation (Fig. 3A, Movie 1, 6/6), as did *MZryr1a* or *MZryr3* larvae (data not shown). In contrast, *ryr1b* larvae failed to exhibit this behavior and simply twitched rapidly (Fig. 3A, Movie 2, 0/6), demonstrating that only RyR1b activity is necessary to perform C-bends in response to stimulation.

To determine whether the RyR channels contributed to normal swimming capabilities, electrically evoked escape responses were recorded and quantified using the DanioVision automated behavioral analysis system (Budick and O'Malley, 2000; Danos and Lauder, 2012) (Fig. 3B). Compared with the average wild-type escape velocity of 45±2.5 mm/s (*n*=20), *ryr1b* animals showed a significant fourfold reduction in their escape velocities (10±1.5 mm/s, *n*=20, *P*<0.0005), consistent with previous observations of the *relatively relaxed* mutant (Hirata et al., 2007). The *ryr1a;ryr1b* double homozygotes completely lacked movement (0±0 mm/s, *n*=10, *P*<0.0005), similar to the effect of complete loss of RyR1 function in the mouse (Takeshima et al., 1994). To summarize, RyR1a and RyR1b channels both contribute positively to functional skeletal muscle contractions in the zebrafish embryo.

### Nonadditive functional interactions among RyR channels

Unexpectedly, *MZryr1a* animals showed a significant increase in their average escape velocities (57±1.6 mm/s, *n*=21, *P*<0.0005) compared with wild-type larvae, indicating that the RyR1a channels function somehow to dampen normal locomotive speed. *MZryr3* animals also showed a small, yet significant, increase in escape velocity (52±1.8 mm/s, *n*=20, *P*<0.005), demonstrating that although the RyR3 channel is not required for muscle contraction, it dampens escape velocity and is required for normal swimming. Consistent with this interpretation, *ryr1a;ryr3* (65±1.5 mm/s, *n*=17) double mutants swam even faster than *MZryr1a* larvae (57±1.6 mm/s, *n*=21, *P*<0.001). Together, these results indicate that both RyR1a and RyR3 channels interact with RyR1b channels to modify swimming behavior.

### Live imaging of Ca^2+^ mobilization reveals fiber type-specific roles for RyR1a and RyR1b and interactions among the RyR channels

**Fig. 4.**
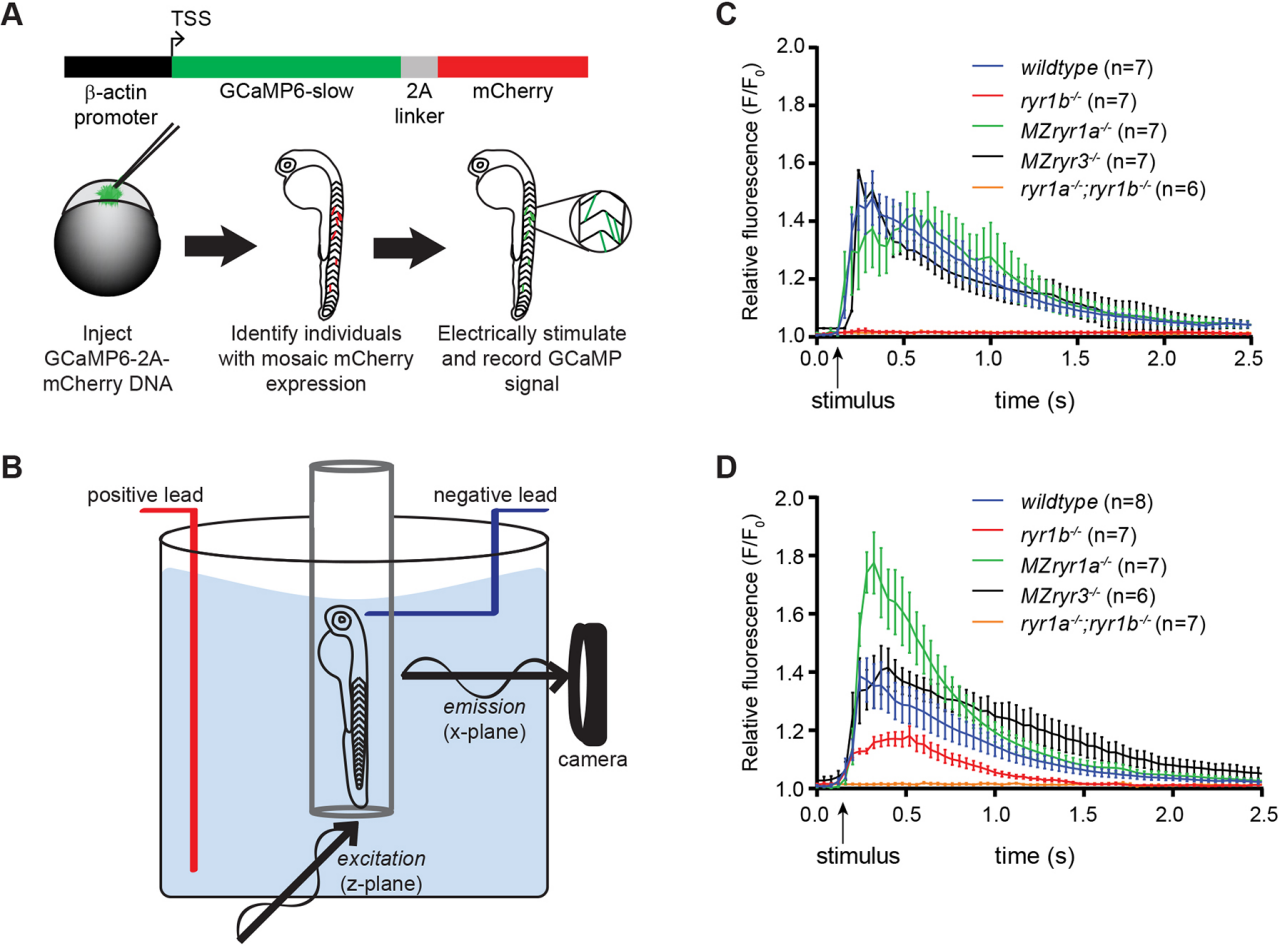
**Muscle fiber type-specific roles of RyR channels.** (A) Schematic for generating zebrafish embryos that mosaically express GCaMP in muscle fibers. One-cell stage embryos were injected with a DNA expression plasmid driving constitutive expression from the B-actin promoter of GCaMP6-slow translationally linked, by a 2A linker peptide, with mCherry. Embryos with isolated muscle fibers that expressed the mCherry reporter were selected for analysis. (B) Experimental setup for SPIM imaging. The 2 dpf embryos, mounted in agarose in a capillary tube, were placed inside an embryo medium-filled chamber. Electrical stimulation to the head was used to evoke a single muscle contraction, and fluorescent GCaMP signal was recorded. (C) GCaMP6-slow signals were recorded from individual fast muscle fibers during a contraction. Loss of RyR1a or RyR3 channels did not alter Ca^2+^ release; however, loss of RyR1b eliminated all Ca^2+^ release in fast fibers. (D) GCaMP6-slow signals were recorded from individual slow muscle fibers during a contraction. Loss of RyR1b activity reduced Ca^2+^ release, whereas loss of RyR1a activity resulted in increased release of Ca^2+^ in slow fibers. Arrows indicate time at which electrical stimulus was delivered. Error bars indicate s.e.m.

Given the unexpected findings that loss of some ryr genes led to increased swimming speeds, we sought to determine how loss of RyR channels affected Ca^2+^ release dynamics in individual slow and fast muscle fibers. RyR-mediated Ca^2+^ release events were recorded in single muscle fibers during an elicited muscle contraction in 2 dpf wild-type or mutant embryos. To visualize single fiber Ca^2+^ release events, a plasmid driving expression of both the Ca^2+^ sensor GCaMP6-slow and the fluorescent reporter mCherry was injected into one-cell eggs. Embryos in which GCaMP and mCherry were expressed mosaically in the myotome were selected for analysis (Fig. 4A). Muscle fiber types were determined based on two criteria: (1) position of the fiber in the myotome, slow being superficial and fast being deep; and (2) orientation of a fiber relative to the horizontal myoseptum, with slow fibers arranged parallel to the horizontal myoseptum and fast fibers approaching it at an oblique angle (Fig. S3). Electrical stimulation was initiated near the head to induce a single, directly evoked muscle contraction; selective plane illumination microscopy (SPIM) was used to record changes in GCaMP signal (Fig. 4B).

RyR1b activity was required for fast-fiber Ca^2+^ release events (Fig. 4C). Fast fibers from wild-type larvae evoked relative signal peaks of ∼1.5-fold above baseline fluorescence (*n*=7). Compared with wild-type fast fibers, the fast fibers of *ryr1b* mutants lacked detectable elevation of GCaMP signal following stimulation (*n*=7, *P*<0.0001) as did the fast fibers of paralyzed *ryr1a;ryr1b* animals (*n*=7, *P*<0.0001). Fast fibers of *MZryr1a* embryos had wild-type levels of Ca^2+^ release (*n*=7, *P*<0.92), consistent with the normal absence of *ryr1a* expression from fast muscle fibers (Fig. 1B,G). *MZryr3* mutant fibers also exhibited no statistically significant changes in either the peak Ca^2+^ release (*n*=6, *P*<0.96) or its decay dynamics (data not shown). Given the expression of *ryr3* in fast fibers (Fig. 1D) and the finding that complete loss of RyR3 resulted in increased swimming velocity of *MZryr3* larvae (Fig. 3), we hypothesize that RyR3 activity had an effect on Ca^2+^ mobilization during a contraction that our methods were insufficient to detect. To summarize, the activity of RyR1b channels is necessary and sufficient for Ca^2+^ release events during a fast fiber contraction. Together with the behavioral analyses, these data indicate that RyR1b channel activity is absolutely required for contraction of fast fibers of the embryo myotome.

The contributions of RyR channels to slow fiber Ca^2+^ release activity are complex (Fig. 4D). Stimulation of skeletal muscle in wild-type (*n*=8) or *MZryr3* (*n*=6) embryos elicited a similar ∼1.4-fold increase in GCaMP signal in slow fibers relative to background fluorescence (*P*<0.91), indicating that RyR3 does not contribute substantially to Ca^2+^ mobilization in these cells. Slow fibers of *ryr1b* embryos displayed significantly reduced, but not absent, GCaMP signals (relative peak at ∼1.2, *n*=7, *P*<0.001). Because *ryr1a;ryr1b* slow muscle fibers had no detectable GCaMP signal in response to stimulation (*n*=7), RyR1a and RyR1b channels both contribute in a positive manner to contraction-associated Ca^2+^ flux in slow muscle. Nevertheless, the role of RyR1a channels in Ca^2+^ release events during muscle contraction is not simply additive. Consistent with the increase in escape velocities exhibited by *MZryr1a* mutant larvae (Fig. 3), the peak levels of Ca^2+^ release in slow fibers of *MZryr1a* embryos significantly exceeded those of wild-type slow fibers during a contraction (relative peak at ∼1.8, *n*=6, *P*<0.05). Thus, the activities of both RyR1a and RyR1b channels are required for wild-type muscle contraction and normal movement; however, in slow fibers, where the two channels are co-expressed, presence of the RyR1a channel attenuates activity of the RyR1b channel through an as-yet-unknown mechanism.

One potential mechanism that could explain the elevated amount of Ca^2+^ release in the slow fibers of *MZryr1a* embryos is genetic compensation by increased expression of a paralogous gene (DeLuna et al., 2010; Kafri et al., 2005; Rossi et al., 2015). We tested whether any of the ryr genes, including those not normally expressed in embryonic muscle, were upregulated in *MZryr1a* embryos at 48 h post fertilization (hpf). Although the abundance of *ryr1a* mRNA was significantly reduced in these embryos, we found no evidence in support of genetic compensation. RT-qPCR analysis indicated that none of the other muscle-expressed ryr genes (*ryr1b* or *ryr3*) were upregulated in mutant embryos. In addition, WISH demonstrated that neither *ryr2a*, which is normally expressed by spinal neurons, nor *ryr2b*, which is normally restricted to cardiomyocytes, were misexpressed in the trunk musculature of mutant embryos (Fig. S5). Compensation by paralogous genes does not explain the augmented Ca^2+^ release and locomotive phenotypes of *MZryr1a* mutants, so we conclude that RyR1a proteins act to modify the Ca^2+^ release dynamics of RyR1b channels.

### RyR activity is required for fiber maturation

**Fig. 5.**
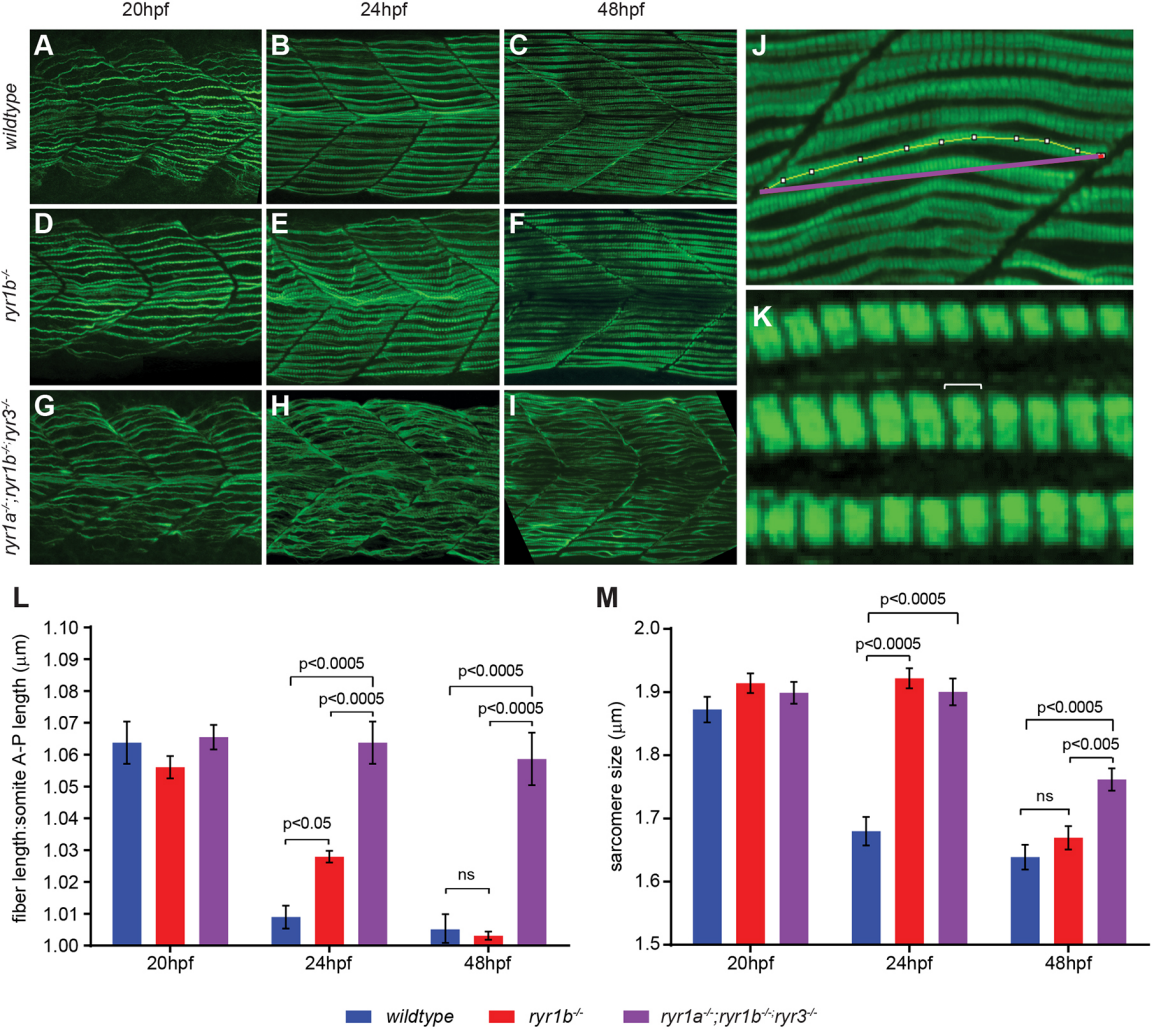
**RyR-mediated muscle contractions are required for slow fiber maturation.** (A-I) Slow fibers were visualized with F59 antibody at 20 and 24 hpf and with the slow fiber-specific S58 antibody at 48 hpf. Slow fibers, which are initially wavy, mature and align with respect to each other as wild-type embryos develop. In *ryr1b* mutant embryos, maturation is delayed; in paralyzed triple mutants, fiber maturation is arrested. (J) Sample image indicating how slow muscle fiber (yellow) and A-P somite (magenta) lengths were determined using ImageJ. (K) Sample image of sarcomere banding indicating how sarcomere A-P lengths (white bracket) were determined. (L) Quantification of slow fiber:somite length ratios. (M) Quantification of sarcomere lengths of slow muscle fibers. One-way ANOVA was used to determine statistical relationships at each developmental time point with Tukey's multiple comparisons test used to adjust *P*-values. To determine fiber length or sarcomere length, five fibers were examined in each of five different embryos for each condition. ns, not significant.

Because abnormal muscle fibers are a common pathological phenotype of RyR1-associated myopathies, we determined whether there were morphological anomalies in the mutant fibers as they first differentiate in embryos. Skeletal muscle fibers undergo a characteristic pattern of maturation following their differentiation, which is thought to be dependent on skeletal muscle activity (Brennan et al., 2005). The maturation of slow fibers, the first muscle fibers to develop in the somite, was visualized from 20 to 48 hpf. As wild-type slow fibers began to differentiate around 20 hpf, they had a ‘wavy’ morphology and exhibited myofibril disorganization (Fig. 5A). With the onset of spontaneous contractions, the fibers quickly straightened and assembled into parallel arrays aligned with the horizontal myoseptum, as seen in 24 or 48 hpf embryos (Fig. 5B,C). Although the slow fibers of paralyzed *ryr1a;ryr1b;ryr3* mutant embryos appeared morphologically similar to wild-type fibers at 20 hpf (Fig. 5G), they failed to straighten by 24 hpf (Fig. 5H). We tested whether developmental delay was sufficient to account for the abnormal fiber morphology seen in 24 hpf paralyzed mutants. Slow fibers were visualized in 48 hpf embryos using the S58 antibody (Fig. 5), which is specific for slow muscle fibers at this later stage (Devoto et al., 1996). In marked contrast to the strict parallel array of superficial slow fibers in wild-type 48 hpf embryos (Fig. 5C), the wavy slow fiber phenotype persisted in triple mutants (Fig. 5I), suggesting that developmental delay was not the source of the morphological abnormalities observed in slow fibers of paralyzed triple-mutant embryos.

To test whether contractile activity correlated with the progression of fiber maturation, we examined slow fiber development in *ryr1b* mutants, which have reduced but not absent Ca^2+^ release in their skeletal fibers (Fig. 4C,D) and diminished swim velocities (Fig. 3). The SSF of *ryr1b* mutants eventually acquire a mature morphology, but with a delayed timetable (Fig. 5D-F). To quantify the straightening process that occurs during normal slow muscle fiber maturation, we determined the lengths of slow muscle fibers relative to the anterior–posterior (A-P) lengths of their somites (Fig. 5J,L). In wild-type embryos, the ratio of fiber length to somite length (fiber:somite) was greater than 1.0 at 20 hpf and approached 1.0 as embryos developed and fibers matured. At 20 hpf, slow fibers of *ryr1b* mutant embryos resembled those of wild-type embryos (Fig. 5A,D,L), but at 24 hpf, *ryr1b* slow fibers still appeared wavy, with an average fiber:somite ratio that was significantly different from that of the wild type (Fig. 5B,E,L). It seemed that slow fibers of *ryr1b* mutant embryos underwent some maturation during this period, as their average fiber:somite length ratio decreased toward a value of 1.0 (Fig. 5L). Indeed, at 48 hpf the arrangement of slow fibers of *ryr1b* mutant and wild-type embryos were indistinguishable and the fibers of both were significantly shorter and straighter than those of *ryr1a;ryr1b;ryr3* mutants (Fig. 5C,F,I,L). These findings are consistent with the interpretation that contractions are required for slow fiber maturation in the developing zebrafish myotome (Brennan et al., 2005).

Because sarcomere size and number determine force generation of a skeletal muscle fiber (Burkholder and Lieber, 2001), we examined whether the altered Ca^2+^ release in mutants affected either of these characteristics (Fig. 5K). At each developmental time point (20, 24, 48 hpf), the numbers of sarcomeres per slow muscle fiber were similar for all three genotypes examined (Fig. S6). In contrast, optimization of sarcomere structure was affected by genotype. Consistent with the developmental pattern of wild-type slow fiber maturation during the 20–48 hpf interval, the average length of a wild-type sarcomere, measured as z-band to z-band distance, shortened progressively during this period (Fig. 5M). Sarcomere dimensions of mutant *ryr1b* and *ryr1a;ryr1b;ryr3* slow fibers were indistinguishable from wild-type embryos at 20 hpf, but the process of sarcomere shortening was altered in the mutants (Fig. 5M). By 24 hpf, sarcomere length was reduced in wild-type embryos but unchanged in *ryr1b* or *ryr1a;ryr1b;ryr3* mutants. By 48 hpf, sarcomeres of *ryr1b* slow fibers had shortened to resemble those of wild-type fibers, but those of paralyzed triple-mutant embryos exhibited only mild reduction in length. Together, these data support the hypothesis that RyR-mediated muscle contractions are required for maturation of sarcomere units and overall slow fiber structure.

### Craniofacial morphogenesis is dependent on RyR-mediated muscle contractions

**Fig. 6.**
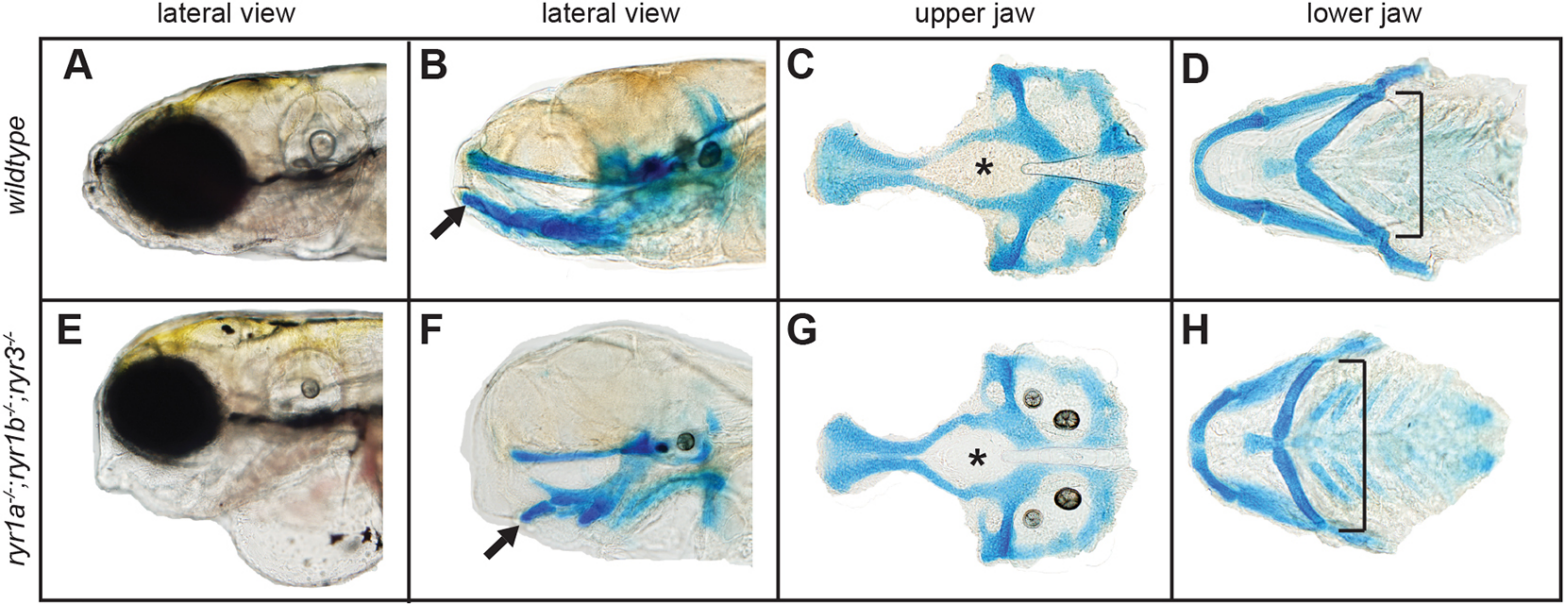
**Paralyzed ryr mutants have abnormal craniofacial morphology.** Craniofacial features of 6 dpf larvae were visualized following staining with Alcian Blue, which marks cartilage. (A-D) Jaw structures of wild-type larvae: lateral view of intact larva (A), lateral view of stained intact larva (B), isolated upper jaw (C) and isolated lower jaw (D). (E-H) Jaw structures of paralyzed *ryr1a;ryr1b;ryr3* larvae: lateral view of intact larva (E), lateral view of stained intact larva (F), isolated upper jaw (G) and isolated lower jaw (H). Jaw architecture of mutant larvae was dramatically shortened in the A-P dimension and wider than normal. Highlighted features include the shape of hypophyseal fenestre (asterisks), the angle between the midline and ceratohyal cartilages of the lower jaw (brackets) and orientation of Meckel's cartilage (arrows).

Individuals with RYR1 mutations may present with an abnormally high-arched palate and additional distinct craniofacial features (Bharucha-Goebel et al., 2013; D'Arcy et al., 2008; Dowling et al., 2011; Jungbluth et al., 2012; Sato et al., 2008; Wilmshurst et al., 2010). Notably, paralyzed *ryr1a;ryr1b* and *ryr1a;ryr1b;ryr3* mutant embryos had somewhat squat and shortened heads relative to wild-type controls (Fig. 6A,B,E,F). Several studies have previously demonstrated a link between skeletal muscle contractions and the establishment of craniofacial architecture (Brunt et al., 2015; Shwartz et al., 2012). To visualize the underlying cartilage-based structures of the head, wild-type and triple-mutant 6 dpf embryos were examined following Alcian Blue staining. All structures of the upper and lower jaw could be distinguished individually, indicating they were properly specified and differentiated; however, the craniofacial architecture of mutant embryos was abnormal. The upper jaw was dramatically shortened in the A-P dimension and wider than normal, as illustrated by alterations in the shape of the hypophyseal fenestre (Fig. 6C,G). The lower jaw had a widened angle between the midline and ceratohyal cartilages and an abnormal ventrally protruding Meckel's cartilage compared with the wild type (Fig. 6B,D,F,H). Thus, in agreement with previous studies of the effects of diminished muscle activity (Brunt et al., 2015; Shwartz et al., 2012), RyR-mediated muscle contractions are required for proper morphogenesis of cartilage-based structures of the head and face of developing zebrafish larvae.

## DISCUSSION

Thus far, it has been difficult to untangle the possible multiple roles of RyR channels in muscle development and disease. Muscle disorders linked to *RYR1* mutations in humans range in clinical presentation from malignant hyperthermia (Chelu et al., 2006; Durham et al., 2008; Yang et al., 2006) to core myopathies (Jungbluth et al., 2011) to congenital fiber type disproportion (Clarke et al., 2010). Severe loss-of-function mutations in the mouse *Ryr1* ortholog can result in paralysis (Takeshima et al., 1994) and dominant mutations in the mouse can affect multiple organs in a syndrome interpreted as developmental delay (Zvaritch et al., 2007). Mutation in the zebrafish *ryr1b* gene results in embryos with abnormal swimming (Hirata et al., 2007). At least three factors confound the interpretation of the phenotypes resulting from mutations in zebrafish genes encoding the RyRs: (1) Mutations can have a range of effects on channel activity or on interactions with co-factors (Van Petegem, 2015). (2) Co-expression of paralogous genes encoding RyRs, each with unknown relative contributions, makes it difficult to know the residual RyR activity in individuals harboring mutations. (3) Little is known about the immediate developmental consequences resulting from RyR loss of function. To provide context for understanding the range of clinical phenotypes that can be associated with mutations affecting the RyR channels, we initiated a study to characterize the embryonic effects of complete loss of function of zebrafish ryr genes. Here, we demonstrate that the channels do indeed contribute to muscle cell-type specification, contractile activity and muscle maturation, helping to explain the heterogeneous set of phenotypes associated with *RYR1* mutations in humans.

Given the recent insight that engineered mutations can be alternatively spliced and, hence, these genes can continue to produce transcripts that lack predicted changes to protein-coding capacity (Anderson et al., 2017; Lalonde et al., 2017; Mou et al., 2017), we tested whether the zebrafish mutant ryr alleles selected for this study were null mutations. We demonstrated that the 34C ‘pan-RyR’ antibody (Airey et al., 1990) was able to detect each of the three RyR channels expressed in the zebrafish myotome. Using this antibody, we showed that homozygotes for each mutant allele failed to express the locus-specific protein product. Moreover, in the two cases tested, mutant transcripts were expressed at greatly diminished levels, a finding consistent with the interpretation that the mutations result in nonsense-mediated decay (Conti and Izaurralde, 2005). Our studies indicate that each mutant is null for its respective protein product. Furthermore, loss of one member of the gene family did not result in altered expression of homologous genes.

All three of the RyR channels expressed in developing zebrafish muscle are required to achieve wild-type Ca^2+^ release dynamics and normal locomotive behaviors. First, each RyR1 channel isoform contributes in a positive manner to muscle contractile activity. Analyses of the *ryr1a;ryr1b* double mutant clearly showed that, as in mammals (Takeshima et al., 1994), complete absence of RyR1 channel activity in zebrafish resulted in paralysis. Consistent with previous study of the *relatively relaxed* allele of *ryr1b*, we found that RyR1b activity was essential for fast-muscle activity in the zebrafish embryo. In contrast to that study, our analyses indicate that RyR1b activity also contributes to Ca^2+^ release in contracting slow muscle fibers. Ca^2+^ release activity in slow muscle fibers was eliminated only when both *ryr1a* and *ryr1b* expression were absent.

The findings that *MZryr1a* mutants had elevated levels of Ca^2+^ release in their contracting slow muscle fibers and exhibited increased escape velocity in response to stimulation demonstrate that the contributions of the RyR channels to muscle activity are not simply independent and additive. The mechanism by which RyR1a dampens the activity of RyR1b is unknown. We tested whether genetic compensation could help explain these observations and found that loss of *ryr1a* did not alter the expression of the other ryr genes. The data are consistent with several hypotheses: (1) RyR1a homotetramers can interact directly with RyR1b homotetramers to dampen their activity. (2) RyR homotetrameric channels compete in some way. (3) RyR1a and RyR1b peptides form heterotetramers that are required for wild-type Ca^2+^ release dynamics. The ability of RyR channels to form heterotetramers has been suggested previously but remains unsubstantiated (Xiao et al., 2002). Additional experiments are required to determine which of these hypotheses is correct.

Our study also lends insight into the roles of RyR3 channels in the zebrafish myotome. Mutant *ryr3* larvae displayed increased escape swimming velocity and loss of RyR3 exaggerated the faster swimming phenotype resulting from loss of RyR1a. Thus, both RyR1a and RyR3 channels can function to dampen the activity of RyR1b. Furthermore, *MZryr3* mutants exhibited enhanced development of Shh-dependent muscle fibers and complete loss of *ryr3* function partially ameliorated the muscle specification defects caused by loss of *MZryr1a*. These results indicate that RyR3 channels appear to be expressed in slow muscle cell precursors (Jurynec et al., 2008). Furthermore, despite our inability to unambiguously detect RyR3 protein in superficial slow muscle fibers, our data are consistent with the interpretation that RyR3 might affect the dynamics of calcium mobilization in overtly differentiated slow muscle cells (Fig. 4D). A previous study pointed to possible functional interactions between RyR channels, finding that RyR3 facilitated the homogeneous distribution of Ca^2+^ release initiated via RyR1 channels in neonatal mouse skeletal muscle fibers (Yang et al., 2001). The channels need not interact physically to affect overall calcium release (Felder and Franzini-Armstrong, 2002). Additional studies are required to determine how the presence of RyR3 and/or RyR1a channels modulates RyR1b channel activity.

Our findings indicate that even modest loss of cumulative RyR channel activity affects fiber-type specification during zebrafish muscle development. Our recent work (Klatt Shaw et al., 2018) indicates that this effect is probably a result of diminished Shh signaling, which informs fiber-type specification in zebrafish. Of relevance to human phenotypes, our work shows that reduced function of subsets of RyR channels can have an effect on muscle development without dramatically altering tissue patterning in general. Congenital fiber-type disproportion is also observed in patients lacking *SEPN1* function (Clarke et al., 2006), whose protein product is needed for RyR channel activity (Jurynec et al., 2008). However, given that patients with mutations affecting tropomyosin also exhibit fiber-type anomalies (Clarke et al., 2008), it is not clear that calcium defects are the sole cause of congenital fiber-type disproportion and there might be multiple pathways that can affect fiber-type development.

It is possible that changes to the numbers of muscle fiber types could contribute to the changes in calcium release levels or escape velocities caused by ryr gene mutations. This result is unlikely, however, because changes in muscle type differentiation do not correlate with the calcium release dynamics or escape velocity changes observed in mutants. For instance, whereas both *MZryr1a* and *MZryr3* mutants have faster escape velocities than wild-type embryos (Fig. 3), *MZryr1a* mutants have reduced numbers of Shh-dependent muscle cells whereas *MZryr3* mutants possess augmented numbers of these cells. Furthermore, *MZryr1a* mutants possess slow muscle fibers that exhibit increased calcium release upon stimulation, but the mutants have a severe loss of Shh-dependent slow muscle.

Recent mouse models designed to recapitulate precisely the human recessive genotypes associated with RyR-related myopathies have revealed that partial loss of function results in diminished muscle strength, fiber hypotrophy and myofibrillar disorganization in the mouse (Brennan et al., 2019; Elbaz et al., 2019). These phenotypes are remarkably similar to those observed in the zebrafish mutants described here. Loss of RyR expression or function in zebrafish embryos led to both diminished skeletal muscle contractility and delayed fiber maturation. Defects in slow fiber maturation and craniofacial architecture probably reflect the relation between skeletal muscle contractions and morphogenic events during early development. Previous work demonstrated that complete elimination of skeletal muscle contractions, by pharmacological treatment or genetic ablation of motor neuron activity, altered slow fiber maturation (Brennan et al., 2005). Our work tested whether there was a correlation between contractile ability and fiber maturation by taking advantage of the intermediate reduction in muscle activity caused by the *ryr1b* mutation. We demonstrated that weak contractions were sufficient for slow fiber maturation, albeit with some temporal delay, lending additional credence to the direct relationship between skeletal muscle contractions and slow fiber development. We note that partial reduction of Shh activation also causes wavy slow muscle fibers (Wolff et al., 2003). However, we found that *ryr1b* mutants had fiber maturation defects in the absence of detectable changes in their numbers of Shh-dependent fiber types. Defects in cell specification could add to the severity of fiber maturation in ryr mutants, but the delayed maturation phenotype cannot be completely explained by Shh specification defects.

Our findings support the hypothesis, put forward in a previous study of paralyzed zebrafish larvae (Shwartz et al., 2012), that muscle contractions help shape craniofacial morphogenesis. The authors proposed that forces generated by muscle contractions pull on underlying cartilage-based structures of the head and are required to shape the overall architecture of the upper and lower jaw, a notion supported by other work that found muscle tension to be required for proper jaw morphogenesis in the zebrafish (Brunt et al., 2015). The relationship between proper craniofacial development and RyR channel activity could help explain the co-occurrence of patients with *RYR1* mutations and high-arched palates, where the palate is unusually high and narrow (Jong et al., 1991; Sato et al., 2008).

In conclusion, our studies reveal that each RyR channel expressed in zebrafish embryonic skeletal muscle has fiber type-specific roles and makes distinct contributions to Ca^2+^ mobilization, muscle fate specification, muscle contraction and swimming behavior. Significantly, our studies demonstrate that, when co-expressed, RyR channels can modify each other's function, resulting in nonadditive contributions to muscle activity. The nonredundant, nonadditive relationships of ryr genes lead us to hypothesize that altered interactions among functionally distinct RyR channels contribute to the complexity of phenotypes that can result from mutations in any one ryr gene.

## MATERIALS AND METHODS

### Fish maintenance

Zebrafish (*Danio rerio*) were maintained in accordance with approved institutional protocols under the supervision of the Institutional Animal Care and Use Committee (IACUC) of the University of Utah, which is fully accredited by the AAALAC. Wild-type zebrafish were from the Tübingen (Tü) strain. The ryr mutations *ryr1a^z42^*, *ryr1b^z43^* and r*yr3^z45^* were generated as described previously (Dahlem et al., 2012; Klatt Shaw et al., 2018). Embryos were collected following natural spawning, maintained at 28.5°C in E3 embryo medium (Westerfield, 1993) and staged (Kimmel et al., 1995).

### Genotyping

Genomic DNA was extracted in 50 mM NaOH at 95°C for 20 min, followed by neutralization with 10% by volume 1 M Tris pH 8. For larvae up to 7 dpf, 50 µl of 50 mM NaOH was used. For adult fish, tail-fin DNA was isolated in 100 µl of 50 mM NaOH. High resolution melt analysis (HRMA) was used to determine genotypes of individual fish as described (Dahlem et al., 2012) using the following primer pairs: *ryr1a* 5′-GTGGACCATTCACCCTGCATC-3′ (forward) and *ryr1a* 5′-TCATCGCCCACTCTGACCTTCT-3′ (reverse); *ryr1b* 5′-GGACCATCCATCCTGCATCCA-3′ (forward) and *ryr1b* 5′-CCTACTCTGACCTTCTCACCCTCGG-3′ (reverse); *ryr3* 5′-GGGAAGCTTGTTGGTGGACAATT-3′ (forward) and *ryr3* 5′-CATCTCCAATGCGCACCTTCT-3′ (reverse).

### WISH, immunohistochemistry and Alcian Blue staining

WISH was performed under standard conditions (Westerfield, 1993). Sequences used as gene-specific probes were generated by RT-PCR using the following primers: *ryr1a* 5′-CCTGCTCATCTCCATGCACCTA-3′ (forward) and *ryr1a* 5′-CAAAGCTCGAATCAGCTCACCC-3′ (reverse); *ryr1b* 5′-GATCAGGGAGAGGAGGAGCG-3′ (forward) and *ryr1b* 5′-ACCTTCACAAGTCCCCAAGAAGA-3′ (reverse); and *ryr3* 5′-CGGTCACGCTATCCTCCTCA-3′ (forward) and *ryr3* 5′-TGAGGACTCCAATCGCTCAAGT-3′ (reverse). Antibodies used for immunohistochemistry (IHC) were obtained from the Developmental Studies Hybridoma Bank and used at the following dilutions: F59 (1:5), S58 (1:5) and 34C (1:5). Prox1 and 4D9 were obtained and used exactly as described (Klatt Shaw et al., 2018). For IHC with S58, 48 hpf embryos were fixed in Carnoy's solution overnight at 4°C (Devoto et al., 1996) and subsequently processed as described (Klatt Shaw et al., 2018). For other antibodies, fixation and washes were performed exactly as described (Klatt Shaw et al., 2018). To detect F59 antibody binding, donkey α-mouse IgG Alexa Fluor 488 secondary antibody (Jackson ImmunoResearch) was used at 1:500. To detect S58, goat α-mouse IgA-FITC secondary antibody (MilliporeSigma) was used at 1:100. For 34C staining, fixed 48 hpf embryos were embedded in OCT compound for cryosectioning as described (Westerfield, 1993); sections (12 μm) were generated using a CM3050 cryostat (Leica Biosystems). For whole-mount 34C staining, 26 hpf embryos were fixed in 4% PFA at room temperature for 1.5 h, washed 3×15 min in PBS containing 1% Triton X-100 (MilliporeSigma) then washed 2×10 min in PBS containing 10% lamb serum. The embryos were subsequently blocked, then treated with 34C at 1:5, following a wash series in PBS containing 10% lamb serum, donkey α-mouse IgG Alexa Fluor 488 secondary antibody (Jackson ImmunoResearch) was used at 1:500. Sectioned tissue was incubated with 34C at room temperature for 3 days in a humid chamber and washed. 34C antibody was detected by incubating for 3 days at room temperature in a humid chamber with donkey α-mouse IgG Alexa Fluor 488 secondary antibody (Jackson ImmunoResearch) used at 1:500. Immunofluorescence images were captured using either a Nikon A1 or an Olympus FV1000 confocal microscope. Alcian Blue (MilliporeSigma) staining was performed as described (Walker and Kimmel, 2007). Individual craniofacial features were manually dissected and imaged with a Zeiss Axioplan compound microscope.

### Intracellular Ca^2+^ recording of individual muscle fibers during a contraction

One-cell stage embryos were microinjected with an expression plasmid comprised of the β-actin promoter constitutively driving the expression of GCaMP6-slow translationally linked via the 2A viral peptide to the mCherry red fluorescent protein. The 2 dpf embryos with isolated mCherry-positive muscle fibers were selected using a M165 C fluorescence stereo microscope (Leica Microsystems) for subsequent experimental procedures. Selected embryos were immobilized in 2% low melt agarose (MilliporeSigma) inside a fluorinated ethylene propylene (FEP) tube (MilliporeSigma). A negative electrical lead was inserted through a small hole in the side of the FEP tube and positioned just above the head of the embryo. The positive lead was placed in the corner of the imaging chamber. An electrical stimulus of 10 mA lasting 50 ms was delivered. To achieve consistent electrical stimulation, the imaging chamber contained high-osmolarity E3 embryo medium (E3 supplemented with NaCl to a final concentration of 15 mM). Imaging was performed using a selective plane illumination microscope (SPIM) (Pitrone et al., 2013) recording at 26.5 frames per second using a 20× water immersion objective. Using ImageJ software, a region of interest box was drawn around an individual fiber and the change in GCaMP fluorescent signal quantified during the course of a contraction. Peak GCaMP signals were compared in order to establish statistical relationships using one-way ANOVA with Dunnett's multiple comparisons test used to adjust *P*-values.

### Behavioral analysis

Movements of 3 dpf larvae were recorded using a DanioVision platform running EthoVision XT (Noldus Information Technology). Escape response was evoked by delivering a whole-field electrical pulse at 10 mA for 500 ms to larvae placed in E3 embryo medium in Falcon six-well flat-bottom plates (Corning). All procedures and recordings were performed at 22.5°C. Larvae were allowed to adjust to environmental conditions for 1 h prior to behavioral analyses. A minimum velocity cut-off threshold of 1.5 mm/s was used.

### Real-time qPCR

Total RNA was harvested from 30 2 dpf embryos homogenized in TRIzol (ThermoFisher Scientific) using a Direct-zol RNA Miniprep Plus kit (Zymo Research). RNA (2 µg total) was used to generate cDNA using SuperScript II reverse transcriptase and oligo(dT)12-18 primers (ThermoFisher Scientific). Luna universal qPCR (New England Biolabs) was used in conjunction with the following primer pairs: *ryr1a* 5′-GATGAAACAGAGCACACTG-3′ (forward) and 5′-CCACATTTATCCAAGCTG-3′ (reverse); *ryr1b* 5′-AAACGGAGCACACAGGA-3′ (forward) and 5′-CCTAAAACAGTCACCAGCAG-3′ (reverse); *ryr2a* 5′-CAACAAGGATGAAACAGAGC-3′ (forward) and 5′-TGGTCCTCGTATTGTTTCC-3′ (reverse); *ryr2b* 5′-CTGTTCTGACCATACTGCG-3′ (forward) and 5′-GGGTCACTTTAAATCCTGGA-3′ (reverse); *ryr3* 5′-ACTGAGCATACCGGTCAG-3′ (forward) and 5′-CCTCATATTGCTTCCGG-3′ (reverse). All experiments were performed in biological triplicate on an Eco Real-Time PCR System running EcoStudy (Illumina). The gene *ef1α* was used as a control gene; primer sequences were obtained from the literature (Karra et al., 2015).

### Statistics

For the single fiber experiments quantifying GCaMP signals, peak GCaMP signals were compared in order to establish statistical relationships using one-way ANOVA with Dunnett's multiple comparisons test used to adjust *P*-values. For the muscle cell-type specification experiments, to calculate the number of cell type-specific nuclei per somite, cells in the last 5 somites over the yolk extension (somites 11-15) were counted and an average value of nuclei per somite determined for each embryo. All embryos (≥8 per genotype) were processed, imaged and quantified in a single experiment. The increase in slow muscle nuclei in *MZryr3* mutants was confirmed in two independent experiments, although data is only shown for a single experiment. Data are represented as mean±s.e.m. Quantifications were completed blind to genotype. One-way ANOVA with Sidak's correction for multiple comparisons was utilized to determine significance. For behavioral analysis experiments, statistical significance was determined using a one-way ANOVA test with Tukey's multiple comparisons test used to adjust the *P*-values. For sarcomere length and number analyses, one-way ANOVA was used to determine statistical relationships at each developmental time point with Tukey's multiple comparisons test used to adjust *P*-values. To determine fiber length or sarcomere length, five fibers were examined in each of five different embryos for each condition.

This article is part of a special collection ‘A Guide to Using Neuromuscular Disease Models for Basic and Preclinical Studies,’ which was launched in a dedicated issue guest edited by Annemieke Aartsma-Rus, Maaike van Putten and James Dowling. See related articles in this collection at http://dmm.biologists.org/collection/neuromuscular.

## Supplementary Material

Supplementary information
