# Supplementary material for: Interactions among ryanodine receptor isotypes contribute to muscle fiber type development and function
Source: Dis Model Mech. 2019 Sep 18;13(2):dmm038844. doi: 10.1242/dmm.038844 (PMC6906632; doi:10.1242/dmm.038844)
Supplement: Supplementary information [file dmm-13-038844-s1.pdf]

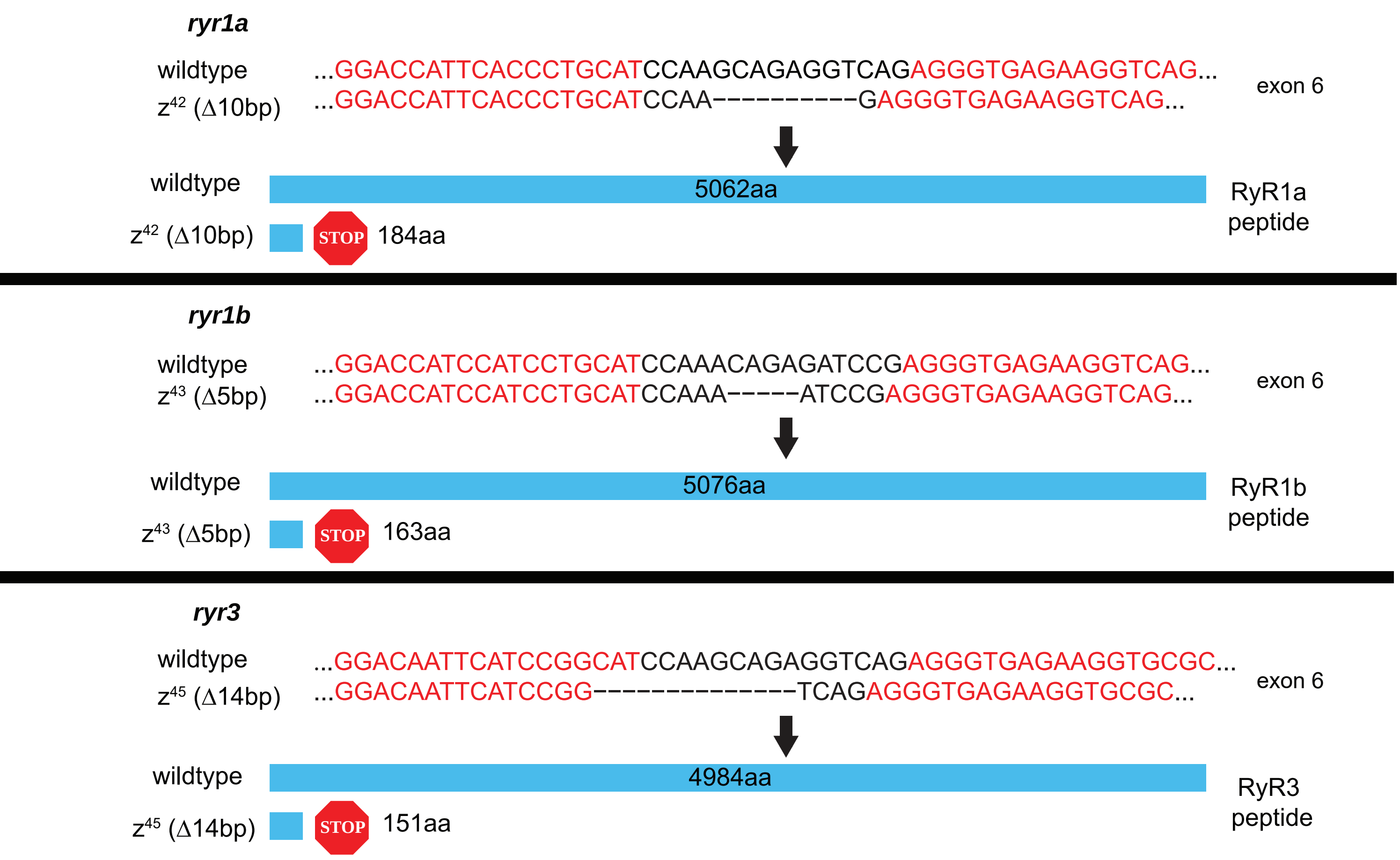

Fig. S1. TALEN-induced *ryr* frame shift alleles.

(A-C) Schematic illustrations of wildtype *ryr* genes and mutant alleles induced by TALEN-stimulated mutagenesis. Sequences from exon 6 of each gene targeted by TALENs are presented. Red letters represent TALEN binding sites and dashes represent deleted nucleotides in the mutant alleles used in this study. Stop signs represent pre-mature stop codons that are predicted to severely truncate the protein product (blue box) of each gene from ~5000 to less than 200 amino acids.

Disease Models & Mechanisms • Supplementary information

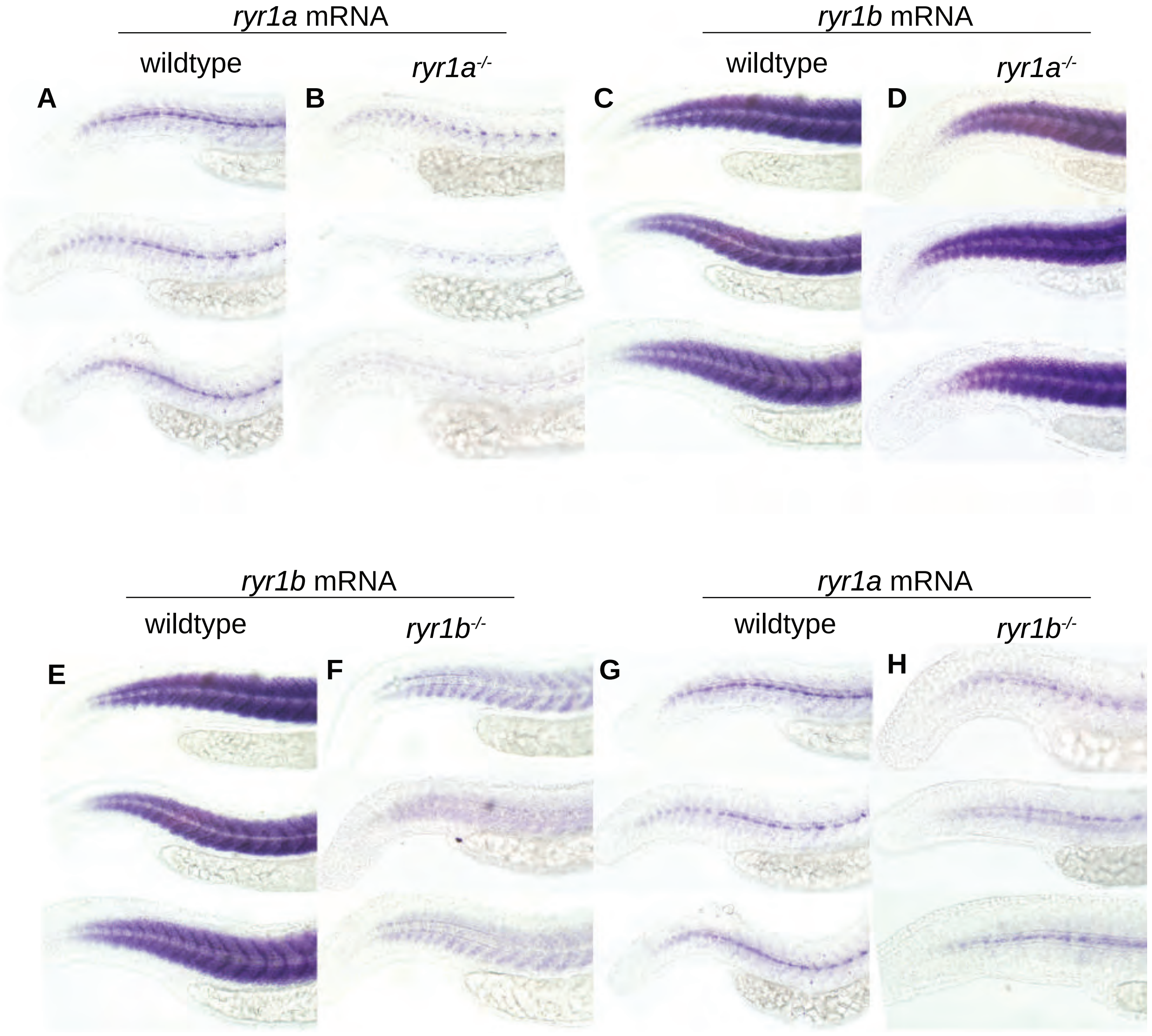

Disease Models & Mechanisms • Supplementary information

Fig. S2. Transcripts derived from *ryr* mutant genes are present at reduced levels. (A-D) Visualization by WISH of *ryr1a* (A,B) or *ryr1b* (C,D) transcripts in the somites of wildtype (A,C) or *ryr1a* mutant (B,D) 24 hpf embryos. The *ryr1a* mutant has reduced steady state levels of *ryr1a* mRNA with normal levels of *ryr1b* expression. (E-H) Visualization by WISH of *ryr1b* (E,F) or *ryr1a* (G,H) mRNAs in the somites of wildtype (A,C) or *ryr1b* mutant (B,D) 24 hpf embryos. The *ryr1b* mutant has reduced steady state levels of *ryr1b* mRNA with normal levels of *ryr1a* expression. Each image is of a different genotyped 24 hpf embryo.

Disease Models & Mechanisms • Supplementary information

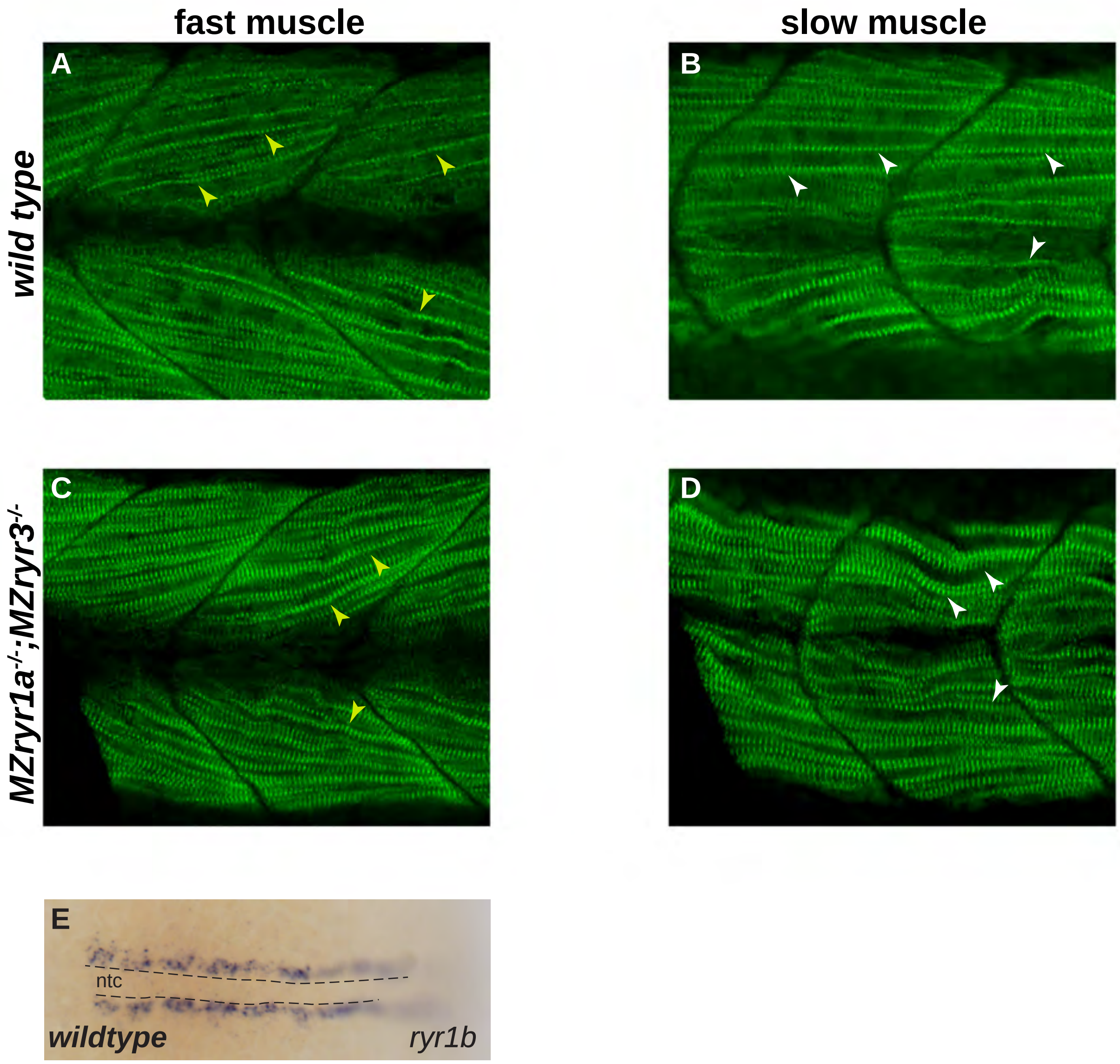
fast muscle
slow muscle
A
B
C
D
wild type
MZryr1a-/-;MZryr3-/-
E
ntc
wildtype
ryr1b

Fig. S3. RyR1b channels are present in slow and fast muscle fibers.

(A-B) Immunohistochemical staining with the pan-RyR 34C antibody reveals the presence of RyR channels at sarcomeres of both fast and slow fibers in wildtype 24 hpf zebrafish embryos. Muscle fibers extend from the anterior to the posterior borders of somites. Fast muscle fibers (A, yellow arrowheads) are deep in the somite and make an obtuse angle with respect to the horizontal myoseptum, whereas slow muscle fibers (B, white arrowheads) are superficial and are aligned parallel to the horizontal myoseptum. (C-D) 34C staining of a *MZryr1a;MZryr3* double mutant embryo demonstrates that RyR1b channels are present in both fast muscle fibers (C, yellow arrowheads) and slow muscle fibers (D, white arrowheads). Panels A and B are images taken at different focal depths of a single WT embryo. Panels C and D are images taken at different focal depths of a single mutant embryo. Anterior is to the left; dorsal is to the top. (E) Visualization of *ryr1b* transcripts in 10 hpf wildtype embryos by WISH. Transcripts are present in adaxial cells, which are adjacent to the outlined notochord (ntc).

Disease Models & Mechanisms • Supplementary information

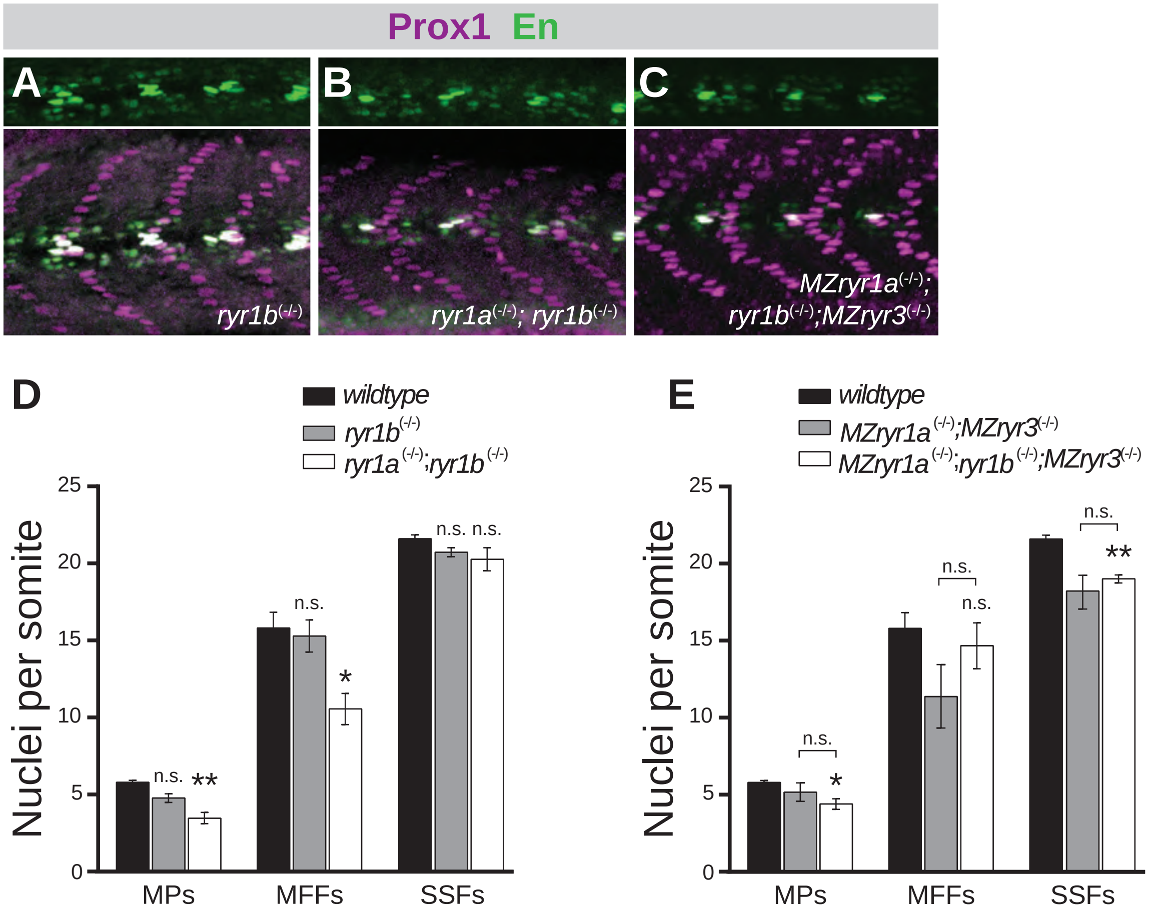
Prox1 En
A
B
C
ryr1b(-/-)
ryr1a(-/-); ryr1b(-/-)
MZryr1a(-/-);
ryr1b(-/-);MZryr3(-/-)
D
wildtype
ryr1b(-/-)
ryr1a(-/-);ryr1b(-/-)
Nuclei per somite
MPs
MFFs
SSFs
E
wildtype
MZryr1a(-/-);MZryr3(-/-)
MZryr1a(-/-);ryr1b(-/-);MZryr3(-/-)

Fig. S4. Formation of Sonic hedgehog-dependent muscle in *ryr1b* mutants.

Muscle cell type patterning in wildtype and mutant zebrafish 24 hpf embryos was assessed by immunohistochemical staining for expression of the Prox1 and Engrailed nuclear proteins. (A-D) Representative images of Prox1 (magenta) and Engrailed (green) staining of somitic muscle in (A) *ryr1b*, (B) *ryr1a;ryr1b*, and (C) *MZryr1a;ryr1b;MZryr3* embryos. Slow muscle pioneer cells (MPs) were identified as cells that expressed both Prox1 and Engrailed antigens; medial fast fibers (MFFs) were identified as cells that expressed only the Engrailed antigen; and superficial slow fibers (SSFs) were identified as cells that expressed only the Prox1 antigen. (D) Quantification of Shh-dependent muscle cell types in WT, *ryr1b*, and *ryr1a;ryr1b* embryos. (E) Quantification of Shh-dependent muscle cell types in *MZryr1a;MZryr3* and *MZryr1a;ryr1b;MZryr3* embryos. One-way ANOVA was used to determine statistical relationships with a Sidak's multiple comparisons test used to adjust p-values. n.s., not significant; *p<0.01; **p<0.001.

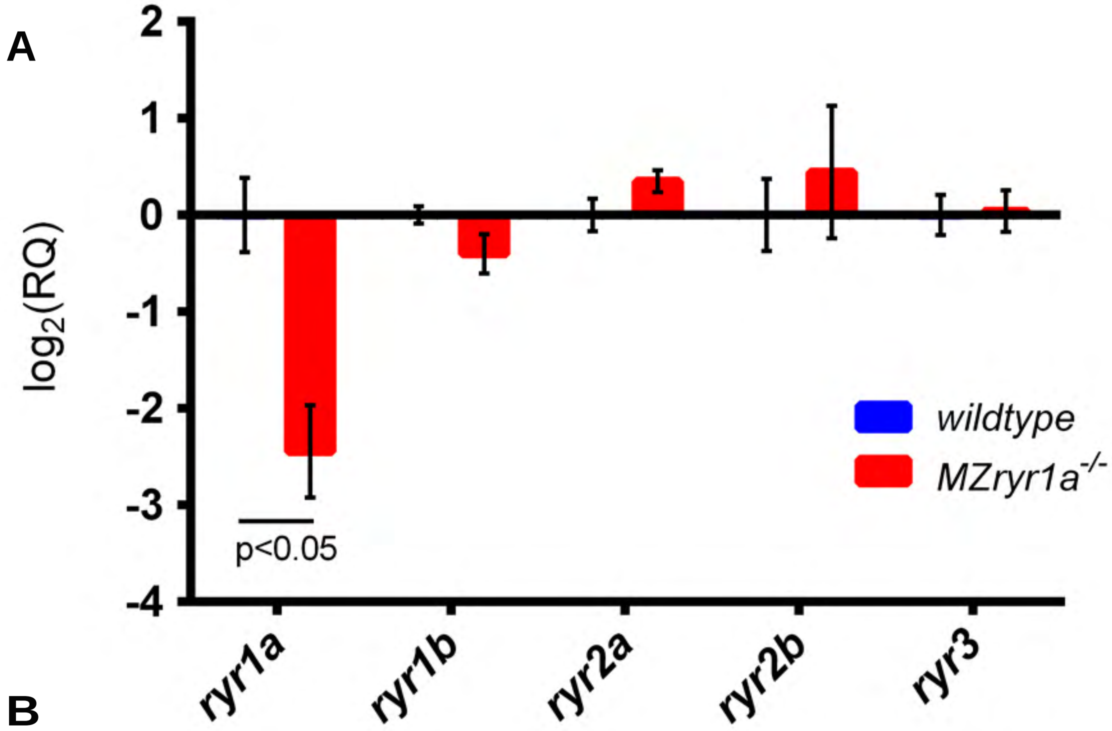
A
2
1
0
-1
-2
-3
-4
log2(RQ)
p<0.05
wildtype
MZryr1a-/-
ryr1a
ryr1b
ryr2a
ryr2b
ryr3

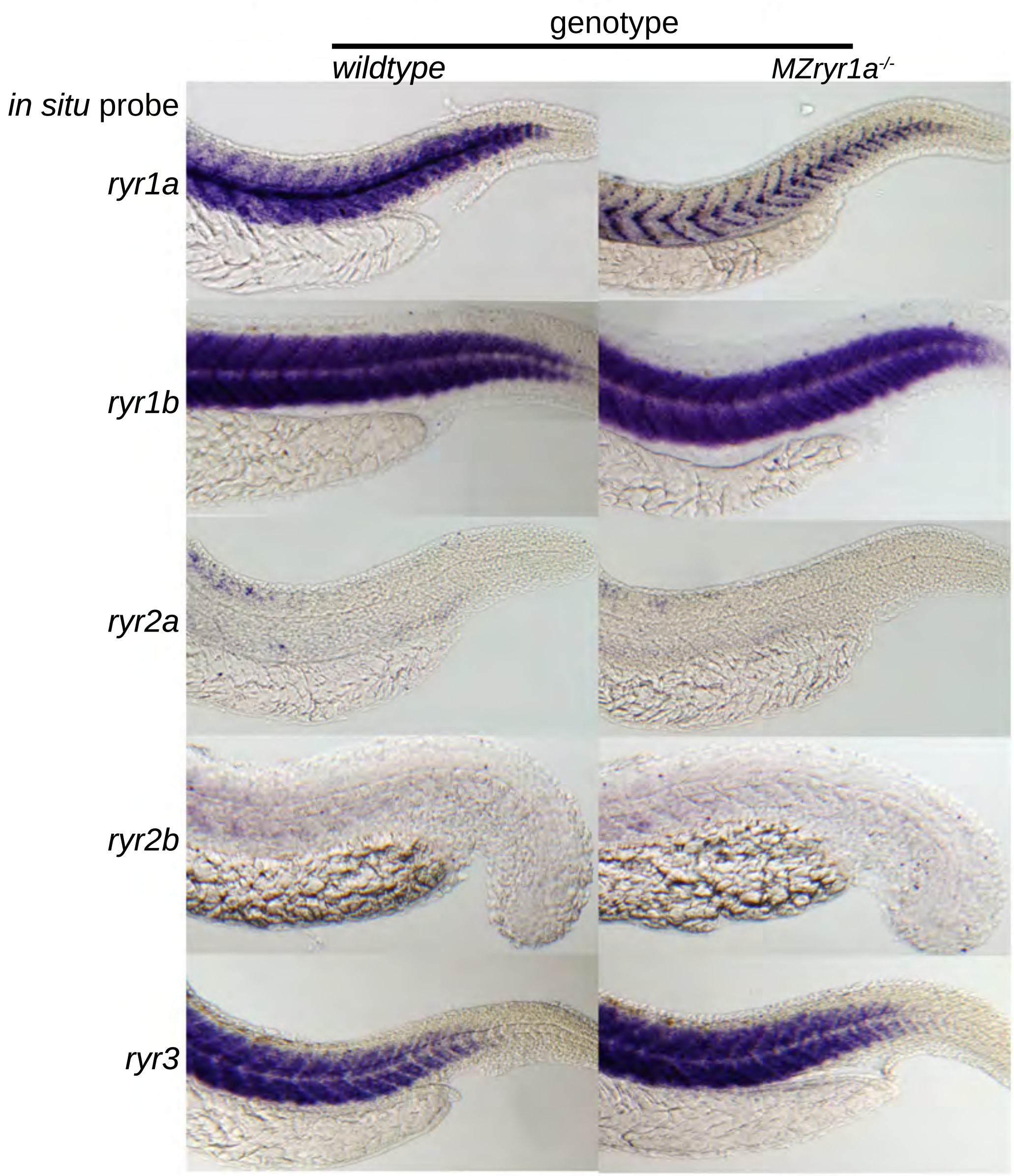
B
genotype
wildtype
MZryr1a-/-
in situ probe
ryr1a
ryr1b
ryr2a
ryr2b
ryr3

Fig. S5. *ryr* genes are neither upregulated nor misexpressed in *MZryr1a* mutants. (A) Relative expression levels of each *ryr* gene transcript in wildtype and *MZryr1a* mutant 48 hpf embryos were determined by RT-qPCR. Transcript levels were normalized to levels of *ef1α* RNA. Expression data were derived from three biological replicate experiments. The abundance of *ryr1a* was significantly reduced in mutants, whereas other *ryr* transcripts were expressed at wildtype levels. The y-axis is a $\log_2$ scale of the relative quantification (RQ) value. Data are presented as the mean +/- standard deviation. Statistical relationships were established using a paired t-test analysis. (B) WISH analysis of *ryr* paralogue expression in 24 hpf *MZryr1a* mutants. The *ryr1a* transcripts are substantially reduced in *MZryr1a* animals, and the expression of the other muscle-specific *ryr* genes, *ryr1b* and *ryr3*, does not appear to be upregulated in mutant embryos. In addition, neither *ryr2a,* which is normally expressed in spinal cord neurons, nor *ryr2b*, which is normally restricted to the heart, assume altered patterns of expression in the mutant embryos.

Disease Models & Mechanisms • Supplementary information

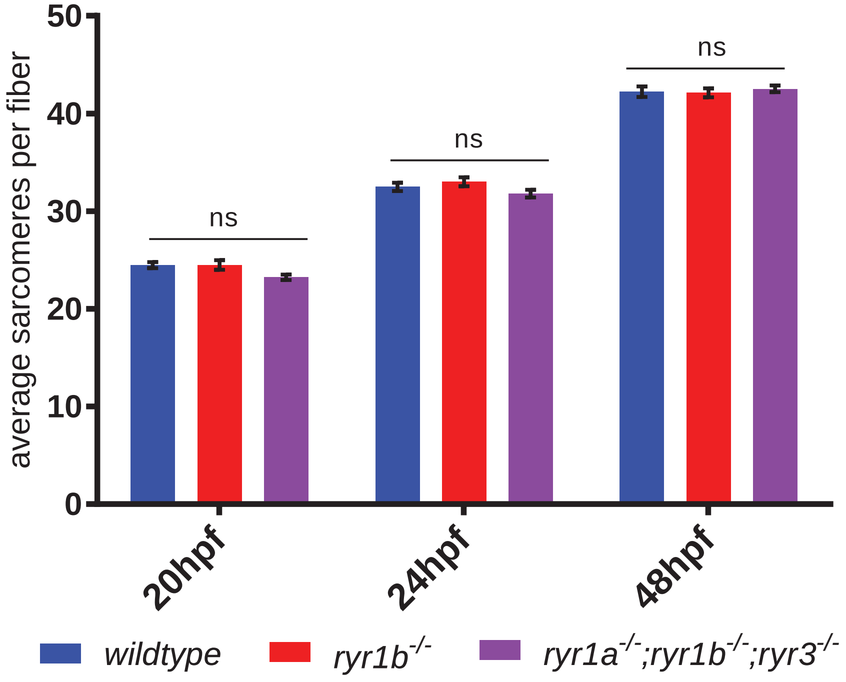

Fig. S6. The sarcomere number is not affected by *ryr* mutations.

Sarcomere banding in slow fibers was visualized with F59 antibody staining at 20 and 24 hpf and with S58 antibody staining at 48 hpf. The average number of sarcomeres per fiber was determined in each genotype at each developmental time point after counting the total number of sarcomeres in five fibers in each of five embryos per condition. One-way ANOVA was used to determine statistical relationships with a Tukey's multiple comparisons test used to adjust p-values, n.s. = not significant.

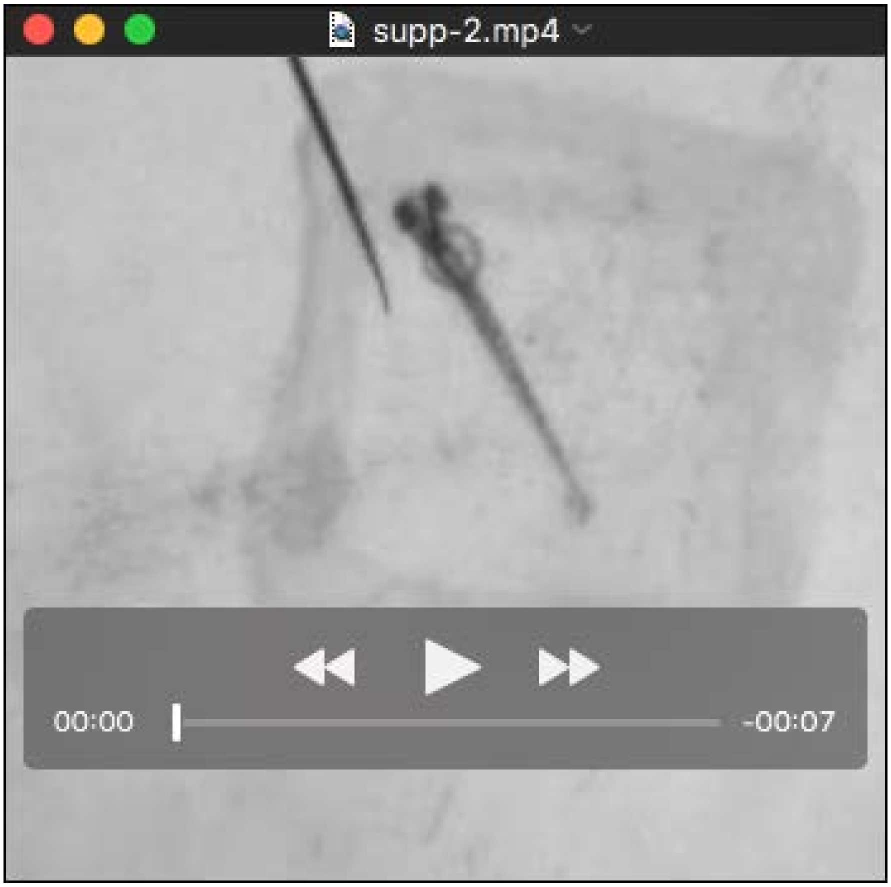

Movie 1. Visualization of wildtype animals displaying C-bend behavior.

Behavioral activities were recorded at 500 frames/sec. Immediately following tactile stimulation to the head, the tail and head bend toward each other forming a 'C' shape. After this initial bend a counter-bend occurs on the opposite side of the animal. The larva then proceeds to swim in a direction that is ~180º from the source of the stimulation.

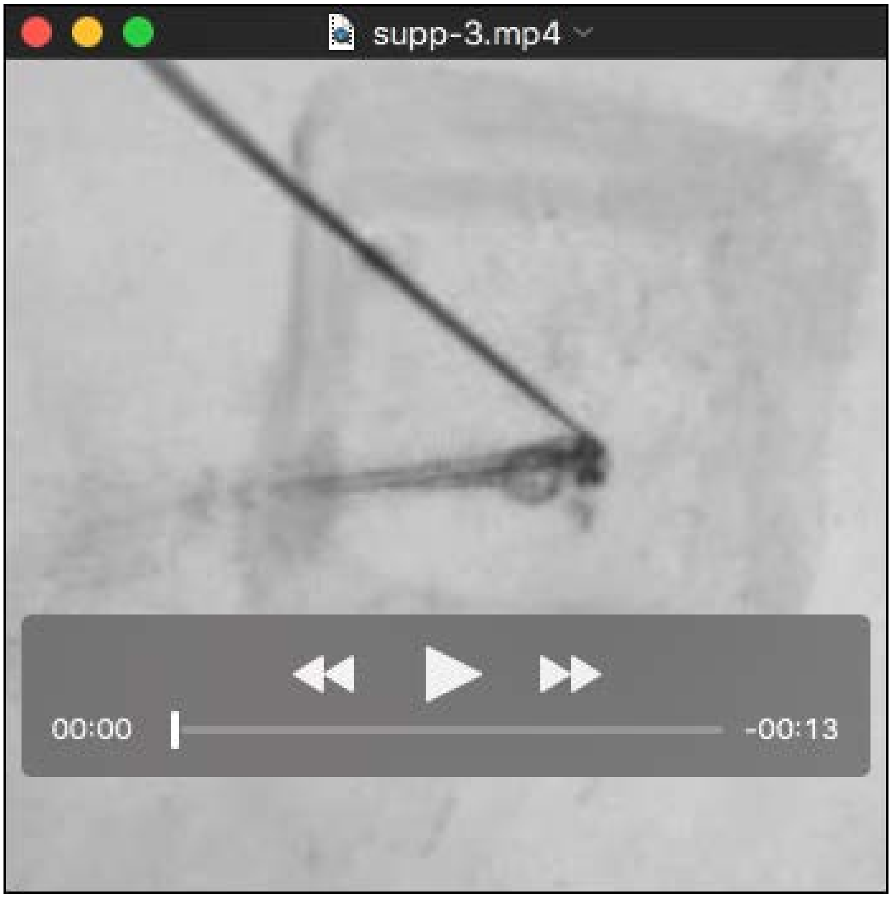

Movie 2. Visualization of *ryr1b*$^{-/-}$ mutants lacking C-bend behavior.

Behavioral activities were recorded at 500 frames/sec. Immediately following tactile stimulation to the head, *ryr1b* mutants reacted with short bursts of weak contractions that result in the larva wiggling in place. These mutants display no C-bend behavior and are unable to swim away from the source of stimulation.

Disease Models & Mechanisms • Supplementary information
